# A functional genomics approach to investigate the differentiation of iPSCs into lung epithelium at air‐liquid interface

**DOI:** 10.1111/jcmm.15568

**Published:** 2020-07-21

**Authors:** Jenny L. Kerschner, Alekh Paranjapye, Shiyi Yin, Dannielle L. Skander, Gurkan Bebek, Shih‐Hsing Leir, Ann Harris

**Affiliations:** ^1^ Department of Genetics and Genome Sciences Case Western Reserve University Cleveland OH USA; ^2^ Systems Biology and Bioinformatics Graduate Program Case Western Reserve University Cleveland OH USA; ^3^ Center for Proteomics and Bioinformatics Case Western Reserve University Cleveland OH USA; ^4^ Department of Nutrition Case Western Reserve University Cleveland OH USA; ^5^ Electrical Engineering and Computer Science Department Case Western Reserve University Cleveland OH USA

**Keywords:** functional genomics, iPSC to HBE‐ALI, open chromatin, transcriptional network, transcriptome

## Abstract

The availability of robust protocols to differentiate induced pluripotent stem cells (iPSCs) into many human cell lineages has transformed research into the origins of human disease. The efficacy of differentiating iPSCs into specific cellular models is influenced by many factors including both intrinsic and extrinsic features. Among the most challenging models is the generation of human bronchial epithelium at air‐liquid interface (HBE‐ALI), which is the gold standard for many studies of respiratory diseases including cystic fibrosis. Here, we perform open chromatin mapping by ATAC‐seq and transcriptomics by RNA‐seq in parallel, to define the functional genomics of key stages of the iPSC to HBE‐ALI differentiation. Within open chromatin peaks, the overrepresented motifs include the architectural protein CTCF at all stages, while motifs for the FOXA pioneer and GATA factor families are seen more often at early stages, and those regulating key airway epithelial functions, such as EHF, are limited to later stages. The RNA‐seq data illustrate dynamic pathways during the iPSC to HBE‐ALI differentiation, and also the marked functional divergence of different iPSC lines at the ALI stages of differentiation. Moreover, a comparison of iPSC‐derived and lung donor‐derived HBE‐ALI cultures reveals substantial differences between these models.

## 1 INTRODUCTION

The technological advances that initiated the field of induced pluripotent stem cell (iPSC) biology^1^, ^2^ provided a new toolbox for examining the establishment of cell identity and the organization of tissues. The availability of stem cells, which upon activation with defined stimuli can produce one of many different human tissue types, has facilitated many aspects of medical research (reviewed in 3). Here, we focus on the epithelium of the human airway that is intimately involved in the aetiology of many common respiratory diseases including chronic obstructive pulmonary disease (COPD), asthma and cystic fibrosis. Among the best resources for investigating human bronchial (HBE) and tracheal (HTE) epithelial cells are primary cultures derived from lung tissue and grown on plastic or differentiated on permeable supports.^4^, ^5^, ^6^ However, these are always in limited supply as they are dependent on the availability of suitable donor organs and tissue. More recently, robust protocols were developed to differentiate iPSCs into lung epithelial cells at air‐liquid interface (ALI), thus modelling the HBE cells on permeable supports.^7^, ^8^, ^9^, ^10^, ^11^, ^12^, ^13^, ^14^, ^15^, ^16^ Extensive characterization of the iPSC‐to‐ALI cell cultures has largely focused on their relevance to disease states (particularly cystic fibrosis ^10^, ^17^) and also generated elegant data on aspects of stem cell biology and the detailed lineages of the cell types.^18^, ^19^, ^20^, ^21^, ^22^, ^23^, ^24^ However, complete maturation of iPSC‐derived ALI cultures into functional HBE‐ALI cells is often challenging. This obstacle may depend not only on the differentiation capacity of individual iPSC lines but also on the absence of appropriate biochemical and/or physical cues.^25^, ^26^, ^27^ Our goal was to develop a robust and unbiased functional genomics pipeline to benchmark the differentiation of iPSC lines from different sources cultured according to diverse protocols in individual laboratories. Here, we utilize genome‐wide methods to examine the gene regulatory networks that drive the pathways of differentiation from iPSCs to ALI cultures and compare the model ALI cultures with HBE‐ALI cells. By intersecting open chromatin data (generated by Assay for Transposase‐Accessible Chromatin using sequencing (ATAC‐seq)^28^) with the transcriptome (generated by RNA‐seq) of defined stages of differentiation of the iPSC to ALI pathway, we reveal the dynamic changes in the transcriptional networks that drive these alterations. We also show the divergence in the molecular signatures of iPSC‐derived ALI and lung donor‐derived HBE‐ALI cells, which may provide insights into the factors that are required to overcome the hurdles of complete maturation of the model ALI cells into mature HBE cells.

## 2 MATERIALS AND METHODS

### 2.1 Cell culture

iPSC lines CR0000008 (Cell line ID: ND2.0), CR0000011 (Cell line ID: ND1.4) were purchased from RUCDR Infinite Biologics https://www.nimhgenetics.org/stem_cells/crm_lines.php (www.rucdr.org). CWRU205 was donated by Dr Paul J. Tesar (Dept. of Genetics and Genome Sciences, Case Western Reserve University). All three lines were from male donors. Differentiation of iPSC to ALI culture was based on the method by Wong et al,^10^ with minor modifications in coating of the membrane inserts (Corning 346012 mm Transwell inserts) for the differentiation protocol. After testing different coating methods described by others,^10^, ^11^ we found Matrigel (Corning 35623, 1/60 dilution) with collagen IV (60 µg/mL) supported the most robust and consistent ALI cultures. The stages of differentiation were confirmed by 1) RT‐qPCR assays specific for NKX2.1, FOXA2, SOX2, SOX9, SOX17, MUC5AC, MUC16, FOXJ1, TP63 and KRT5 transcripts, using primers shown in Table S1; and specific antibodies to CXCR4 (CD184, BD Pharmigen 5559740), SOX17 (BD Pharmigen 562594), NKX2.1 (TTF1; Abcam ab76013), SOX2 (GeneTex GTX101507), FOXA2 (Abcam ab60721) for FACS at DE and AFE stages; and immunofluorescence for NKX2.1, FOXA2, SCGB1A1 (CC10, Santa Cruz Biotechnology (SCB) sc‐365992) and MUC5B (SCB sc‐21768) at AFE, LP3a and ALIw5 stages.

Donor‐derived HBE cells were obtained from the Marsico Lung Institute CF Center Tissue Procurement and Cell Culture Core (University of North Carolina (UNC), Chapel Hill, NC) and cultured according to the published protocols^5^, ^6^ in accordance with relevant guidelines. The cells were obtained under protocol #03‐1396 approved by the University of North Carolina at Chapel Hill Biomedical Institutional Review Board. All donors or their authorized representatives provided informed consent for research use of explanted lungs. This work was also approved by the Case Western Reserve University Institutional Review Board.

### 2.2 RNA preparation, reverse transcription quantitative PCR (RT‐qPCR) and RNA‐seq

Total RNA was extracted from iPSCs at different stages of differentiation using TRIzol (Invitrogen) using their protocol. RT‐qPCR was done using our standard protocols^29^ with primers shown in Table S1. RNA‐seq (SR 50bp) was performed as described previously.^30^

### 2.3 ATAC‐seq

ATAC‐seq was performed on 50 000 cells as previously described^28^ with the following minor modifications. The initial cell pellet was washed 1‐2 times in 100 μL cold PBS and centrifuged for 5 minutes at 500 × *g* at 4°C. The washed cell pellet was resuspended in 100 μL cold lysis buffer (100 mmol/L Tris‐Cl, pH 7.4; 10 mmol/L NaCl; 3mmol/L MgCl_2_; 0.1% (v/v) IGEPAL CA‐630, freshly prepared), incubated on ice for 10 minutes and centrifuged for 10 minutes at 500 × *g* at 4°C. The nuclear pellet was washed once in 50 μL homemade TD buffer^31^ (20 mmol/L Tris(hydroxymethyl)aminomethane; 10 mmol/L MgCl_2_; 20% (v/v) dimethylformamide) and centrifuged for 5 minutes at 500 × *g* at 4°C, before proceeding with tagmentation using Nextera Tn5 Transposase and commercial 2X TD buffer (Illumina). Following preparation, the final ATAC‐seq libraries were purified with Agencourt AMPure XP magnetic beads (Beckman‐Coulter) at a sample to bead ratio of 1:1.2, following the manufacturer's protocol, and eluted into 20 μL Buffer EB (Qiagen). ATAC‐seq library size distributions were visualized by TapeStation (Agilent) and quantified using the KAPA Library Quantification Kit (Roche). Six to seven libraries were pooled, per lane, and sequenced on a HiSeq4000 (Illumina) using 100 bp paired‐end sequencing.

### 2.4 RNA‐seq analysis

Raw reads were aligned with STAR 2.6 (https://github.com/alexdobin/STAR).^32^ Aligned reads were then assigned to genomic features with featureCounts version 1.6.3 in the Subread package (http://subread.sourceforge.net/)^33^ and differential gene expression was analysed using DEseq2 version 1.22.1 (https://www.bioconductor.org/packages/release/bioc/html/DESeq2.html).^34^

Raw reads of six untreated healthy donor‐derived HBE‐ALI samples were acquired from the GEO database Series GSE97036.^35^ Data sets were processed using the same pipeline. The cells used in this series were as described above for donor‐derived HBE cells.

### 2.5 Gene ontology analysis

Differentially expressed genes were filtered to enrich for those with a fold change ≥1.5 and Benjamini‐Hochberg adjusted *P*‐value ≤ 0.01. Gene lists were read into the PANTHER gene ontology database (http://pantherdb.org/)^36^ using the biological processes term. Results were further validated using gProfiler. Statistically significant results were filtered for categories passing a *P*‐value of 0.001 with the Bonferroni correction for multiple testing.

### 2.6 ATAC‐seq data processing

Raw reads from the HiSeq4000 were processed using the ENCODE ATAC‐seq pipeline (Dec 2018)(https://github.com/kundajelab/atac‐seq‐pipeline). Replicates were used to generate peaks with an IDR threshold of 0.5. Analysis of enriched motifs within the IDR peaks was performed with HOMER (4.7.2q) (http://homer.ucsd.edu/homer/index.html).^37^ PCA and heatmaps were generated by DiffBind (2.10.0) (http://bioconductor.org/packages/release/bioc/vignettes/DiffBind/ inst/doc/DiffBind.pdf).^38^

### 2.7 Binding and expression target analysis BETA

Differential gene expression for STAR aligned reads was analysed using the Cufflinks suite v2.2.1. (http://cole‐trapnell‐lab.github.io/cufflinks/).^39^ Cuffdiff output for pairwise comparisons was processed against ATAC‐seq peak files for each stage in the pair independently using the BETA software version 1.0.7 (http://cistrome.org/BETA/)^40^ to determine peak distribution at differentially expressed genes (DEGs). Peaks were chosen within 20 kb from the gene body, and function prediction was determined by the rank product of the estimated regulatory potential of factor binding and change in expression of the gene target.

### 2.8 Immunofluorescence

iPSC ALI cultures (week 5) on membrane filters were harvested and fixed in 4% paraformaldehyde (in PBS), embedded in paraffin and cut into 5‐μm sections. After deparaffinization and rehydration, antigen retrieval was performed by heating with citric acid (10 mmol/L, pH 6.0) in a 98°C water bath for 45 minutes. The tissue sections were subsequently washed 3 times in blocking solution (1% BSA and 0.05% Saponin in PBS). The sections were incubated with the primary antibodies (diluted in PBS containing 0.1% BSA) overnight at 4°C. The sections were then washed 3 times in PBS containing 0.05% Tween 20, followed by incubation with secondary antibodies (diluted in PBS with 0.1% BSA) for 1 hour at room temperature. After 3 washes and counterstaining with 4,6‐diamidino‐2‐phenylindole (DAPI), the samples were mounted with ProLong Gold Antifade Mountant (Invitrogen). The specimens were imaged on a fluorescence microscope (Leica DM 6000). Primary antibodies used were as follows: NKX2.1 (TTF1; Abcam ab76013), FOXA2 (Abcam ab60721) and CC10 (SCB sc‐365992). Secondary antibodies used were as follows: Rhodamine Red™‐X (RRX) AffiniPure Donkey Anti‐Mouse IgG (H + L) (Jackson ImmunoResearch 715‐295‐151) and Alexa Fluor 488 (AF488) AffiniPure Donkey Anti‐Rabbit IgG (H + L) (Jackson ImmunoResearch 711‐545‐152).

## 3 RESULTS

**Figure 1:**
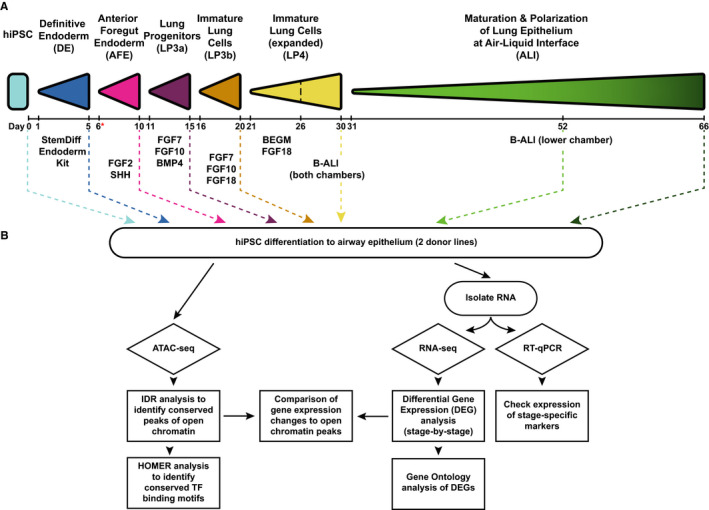
Schematic to show the protocol used to differentiate iPSC into lung epithelial cells at air‐liquid interface (ALI) and the experimental pipeline. A, Summary of protocol and timeline for iPSC to ALI differentiation. The same colour‐coding is used throughout the figures to denote each stage of differentiation. The red * at day 6 denotes when cells are transitioned to permeable supports. B, Workflow of experimental procedures and bioinformatics analysis of data for each experiment

Our experimental and analysis pipelines are summarized in Figure 1. In initial experiments, we differentiated three iPSC lines, ND2.0, ND1.4 and CWRU205, which came from 2 different sources. We followed the standard protocols as described in the methods section to culture the iPSCs and differentiate them into airway epithelial cells at ALI (Figure 1A). Cells were collected for ATAC‐seq and RNA‐seq at 6 time‐points: definitive endoderm (DE), anterior foregut endoderm (AFE) and lung progenitor cells 3a, 3b (LP3a, LP3b), at air‐liquid interface after weeks 3 and weeks 5 (ALIw3 and ALIw5). The ND1.4 iPSC line repeatedly failed to differentiate fully to the ALI stage (either through not adhering to membrane supports at the transition to AFE cells, or due to reduced cell proliferation starting at LP3a), so this line was not analysed further. Open chromatin and RNA‐seq data were derived from cells in multiple differentiation processes of the ND2.0 and CWRU205 lines. However, data presented here are from one differentiation process where these 2 lines were grown in parallel using the same batches of culture media, supplements and culture inserts, to minimize variation that was not inherent to the cells. The RNA‐seq and ATAC‐seq pipeline are summarized in Figure 1B.

### 3.1 Open chromatin mapping through differentiation from iPSC‐derived definitive endoderm through to air‐liquid interface lung epithelial cell cultures

**Figure 2:**
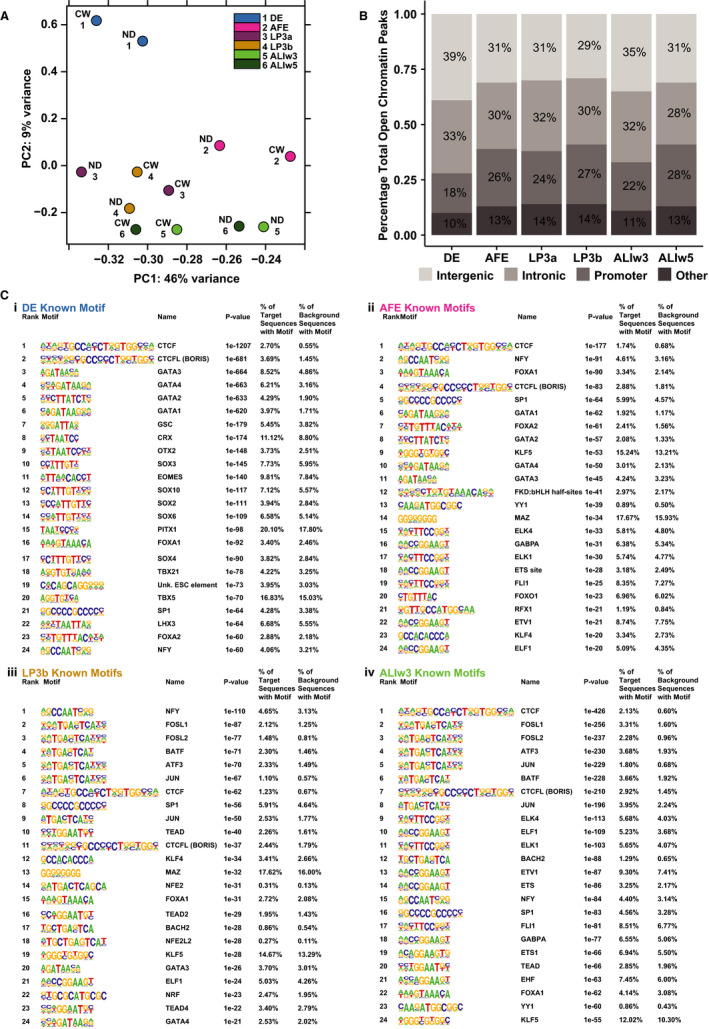
Analysis of open chromatin through iPSC to ALI differentiation. A, Principal component analysis (PCA) plot showing relationships between open chromatin at different stages of differentiation from the ND2.0 (ND) and CWRU205 (CW) iPSC lines. Each developmental stage is colour coded and numbered according to the inset panel. B, Genomic distribution of open chromatin peaks at each stage of differentiation from an IDR analysis of the ND2.0 and CWRU205 iPSC lines. Peaks called as: intergenic, intronic, promoter (defined as 2 kb upstream of the transcription start site) or other. C, HOMER analysis showing overrepresented binding motifs for known transcription factors under open chromatin peaks at 4 developmental stages i) DE, ii) AFE, iii) LP3b, iv) ALIw3

We first used ATAC‐seq to identify the regions of open/active chromatin at each developmental stage and combined data from the ND2.0 and CWRU205 lines to generate a reproducible peak set by irreproducible discovery rate (IDR) analysis. Principal component analysis (PCA) (Figure 2A) showed close concordance between the open chromatin profiles of the two donors at each time‐point from DE to LP3b. In contrast, at the ALIw3 and ALIw5 stages the within donor similarities were greater than time‐point–associated features. Of note, the open chromatin profiles of the DE stage were most divergent from all other stages of differentiation. We also examined the IDR peak distributions across the different genomic regions (intergenic, intronic, promoter [within 2 kb 5’ of the transcriptional start site] or other) and found these to be largely consistent between differentiation stages (Figure 2B). Next, we performed transcription factor (TF) motif enrichment analysis on the IDR peaks at each stage of differentiation using HOMER (Figure 2C, Figure S1). Focusing on known motifs, many factors are significantly enriched in peaks of open chromatin including binding sites for architectural proteins CCCTC‐binding factor (CTCF)^41^ and Brother of the Regulator of Imprinted Sites (BORIS),^42^ also known as CCCTC‐binding factor‐like (CTCFL). CTCF is the most overrepresented motif at DE, AFE and ALIw3 stages (Figure 2C i, ii, iv). Consistent with its pivotal role in organizing higher order chromatin structure genome‐wide, the CTCF motif is among the top 7 motifs at all stages of differentiation (Figure 2C, Figure S1). Among TFs with pioneer activity (opening chromatin to increase accessibility for other TFs) and also with key roles in development, motifs for GATA‐binding proteins 1‐4 (GATA1‐4)^43^, ^44^, ^45^, ^46^ and forkhead box A1 and A2 (FOXA1/A2)^45^, ^46^, ^47^ are highly overrepresented in open chromatin at DE and AFE stages (Figure 2Ci‐ii). Motifs for many other known developmental factors, including orthodenticle homeobox 2 (OTX2),^48^, ^49^ the T‐box factor eomesodermin (EOMES)^50^ and multiple SRY‐box transcription factors (SOX2, 4, 6 and 10),^51^ are overrepresented only in DE cells (Figure 2Ci). Krüppel‐like factor 5 (KLF5) and forkhead box O1 (FOXO1) motifs are highly overrepresented in AFE cells but not DE, consistent with the role of these TFs in airway differentiation and function.^52^, ^53^, ^54^ At the LP3b and ALIw3 stages, among the most overrepresented motifs are members of the Fos and JUN families, which dimerize to form AP‐1, a TF with important roles in differentiation.^55^ FOXA1 and KLF5 motifs also remain enriched at these stages consistent with an important role of both factors in airway epithelial biology.^52^, ^56^ Motifs for many ETS factors, including ETS homologous factor (EHF), ETS Proto‐oncogene 1, transcription factor (ETS1), ETS transcription factor ELK1 (ELK1) and ETS transcription factor ELK4 (ELK4), become highly overrepresented as the cells differentiate into ALI cultures.

### 3.2 The transcriptome through differentiation from iPSC‐derived definitive endoderm through to air‐liquid interface lung epithelial cell cultures

**Figure 3:**
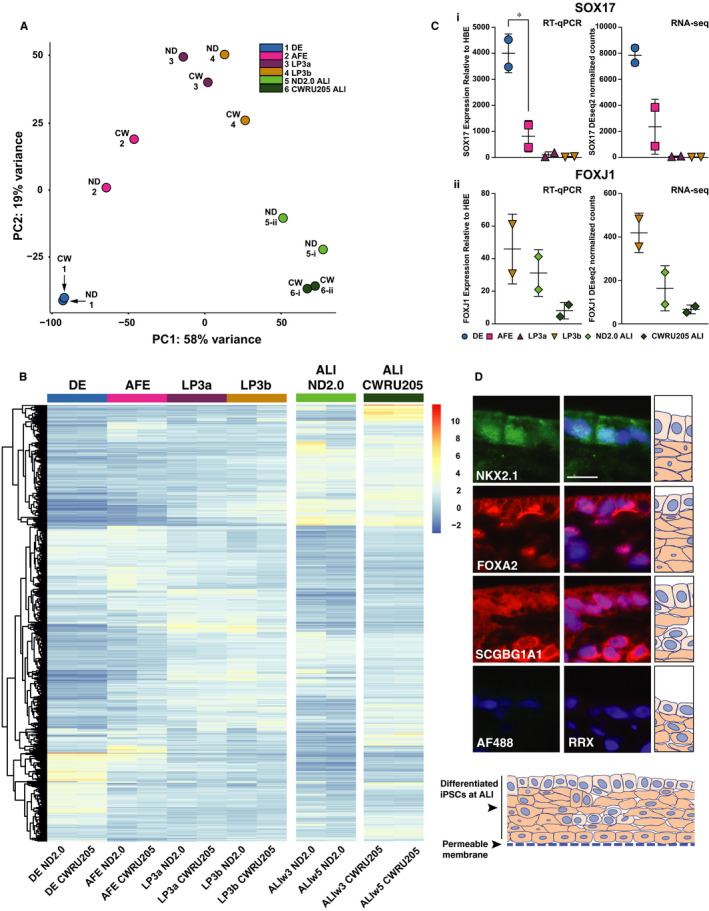
Gene expression profiles of iPSC differentiation to ALI. A, PCA for differentially expressed genes at each time‐point from DE to ALIw5 in ND2.0 (ND) and CWRU205 (CW) iPSC lines. ND2.0 and CWRU205 iPSC lines are used as biological replicates for each developmental stage from DE to LP3b. Due to substantial divergence between the 2 lines at ALI, week 3 and week 5 ALI for each line are used as replicates for ALI, with i denoting ALIw3 and ii denoting ALIw5 samples. B, Heatmap of 1000 differentially expressed genes, normalized for sequencing depth across all samples. C. Left panels, RT‐qPCR of SOX17 (i) and FOXJ1 (ii) normalized to the housekeeping gene PGK1 and shown relative to an average of 3 HBE cultures grown on plastic. * denotes *P* < 0.05 using an unpaired t test comparing sequential stages. Right panels, corresponding RNA‐seq DEseq2 normalized counts. D. Immunofluorescence detection of NXK2.1, FOXA2 and SCGBG1A1 in iPSC ND2.0‐derived ALIw5 cultures. For each marker the bottom panels show no primary antibody negative controls (AF488 donkey anti‐rabbit IgG and RRX donkey anti‐mouse IgG) with nuclei stained with DAPI. Upper panels show AF488 staining of NKX2.1, RRX staining of FOXA2 and SCGBG1A1/CC10 on the left and merged images with DAPI (nuclei) on the right. The magnification is the same for all images and the size bar = 10 µm. A diagram below the fluorescent images represents the multilayered differentiated iPSC cells grown on a permeable membrane and cartoons on the right orientate the images with respect to this diagram

To compare temporal changes in gene expression, RNA‐seq was performed at each stage of differentiation in the ND2.0 and CWRU205 iPSC lines. Gene expression values were quantified using the featureCounts software in the Subread package (r3.1) and normalized for sequencing depth and RNA composition with DESeq2. The PCA plot in Figure 3A shows a good correlation between the samples from the two lines at each stage until the ALI cultures. Normalized count distributions for the two iPSC lines consistently clustered together across all principal components. Once the cells were exposed to ALI conditions, each line remained more similar to itself between 3 and 5 weeks at ALI than to the other line at the same time‐point. To accommodate the variance between the two lines at the ALIw3 and ALIw5 stages, results for each time‐point were used as biological replicates. Following adjustment for sequencing depth between samples, we identified 8940 differentially expressed genes (*P* < 0 .005) at one or more stage in the full developmental pathway from DE to ALIw5. A heatmap of the expression profile matrix for the top 1000 differentially expressed genes (DEGs) revealed several of these trajectories including genes most up‐regulated in the early stages or those that are ALI‐specific (Figure 3B). Among the most significant DEGs, passing a *P*‐value threshold of 0.005, in DE compared to all other stages are Cerberus 1, DAN family BMP agonist (*CER1*), a cytokine that binds directly to bone morphogenetic proteins and inhibits their activity^57^, ^58^ and Left‐Right Determination Factor 1 (*LEFTY1*), which encodes a transforming growth factor‐beta (TGFβ) family member (Table 1). LEFTY1 regulates LEFTY2 and NODAL and is required for left‐right axis formation during development.^59^ Expression of both *CER1* and *LEFTY1* at later stages of differentiation has a log_2_ fold difference of −1.93 compared to DE. In contrast, among the most significant DEGs in the combined ALIw3 and ALIw5 samples compared to other stages of differentiation are the two subunits of sodium/potassium Na+/K+ ATPase (*ATP1A1* and *ATP1B1*), which together are essential for the active transport of Na+ and K+ across cell membranes (Table 2). Also more highly expressed in ALI cultures is the long non‐coding RNA Metastasis‐Associated Lung Adenocarcinoma Transcript 1 (*MALAT1*) and two members of the Serpin family of protease inhibitors *SERPINA1* and *SERPINA3* (Table 2), which have a log_2_ fold change of 1.87, 7.57 and 9.08, respectively, compared to the earlier stages. A key target of SERPINA1 (alpha 1 antitrypsin) is elastase, and defects in the protein are associated emphysema.^60^

**Table 1:** Most significant DEGs in DE compared to all other stages of differentiation

Gene^a^	log_2_ (FoldChange)	log_2_ (FC) std. error	Wald statistic	*P*‐value	*P*.adjusted^b^
CER1	−1.93	0.59	−3.29	1.01E‐03	3.40E‐03
LEFTY1	−1.93	0.52	−3.72	2.01E‐04	8.09E‐04
SLC2A3	−5.68	0.66	−8.66	4.61E‐18	1.31E‐16
LAPTM4B	−1.89	0.46	−4.11	4.03E‐05	1.90E‐04
ERBB4	−6.29	0.75	−8.37	5.71E‐17	1.44E‐15
RHOBTB3	−1.73	0.43	−4.00	6.31E‐05	2.85E‐04
PODXL	−5.24	0.50	−10.51	7.61E‐26	4.30E‐24
CXCR4	−5.24	0.35	−14.93	2.04E‐50	6.81E‐48
LARP7	−1.09	0.25	−4.41	1.03E‐05	5.49E‐05
SFRP1	−4.97	0.36	−13.86	1.06E‐43	2.00E‐41
SERPINE2	−3.22	0.35	−9.27	1.91E‐20	6.82E‐19
LIN28A	−9.60	0.32	−30.14	1.50E‐199	3.06E‐195
OTX2	−6.66	0.59	−11.33	9.59E‐30	7.62E‐28
TDGF1	−7.13	0.67	−10.66	1.54E‐26	9.29E‐25
CYP26A1	−5.24	0.73	−7.18	6.74E‐13	1.06E‐11
SLC2A14	−5.01	0.59	−8.46	2.79E‐17	7.28E‐16
FZD7	−1.80	0.44	−4.11	3.94E‐05	1.86E‐04
SCD	−1.26	0.30	−4.13	3.58E‐05	1.71E‐04
CCNG1	−1.34	0.21	−6.29	3.24E‐10	3.62E‐09

^a^ Genes are presented in order of high to low by subtracting the average DEseq2 normalized counts from all other stages from the average DEseq2 normalized counts for DE samples (ND2.0 and CWRU205).

^b^ Adjusted *P*‐value is calculated with the Benjamini‐Hochberg procedure.

**Table 2:** Most significant DEGs in aggregated ALIw3/w5 compared to all other stages of differentiation

Gene^a^	log_2_ (FoldChange)	log_2_ (FC) std. error	Wald statistic	*P*‐value	*P*.adjusted^b^
ATP1A1	2.80	0.36	7.84	4.35E‐15	8.99E‐14
MALAT1	1.87	0.40	4.70	2.58E‐06	1.56E‐05
COL1A2	4.05	0.75	5.39	7.14E‐08	5.64E‐07
SERPINA1	7.57	0.55	13.66	1.67E‐42	3.01E‐40
ITGB1	1.95	0.36	5.36	8.29E‐08	6.50E‐07
CEACAM6	8.09	0.95	8.54	1.38E‐17	3.72E‐16
B2M	4.12	0.56	7.36	1.77E‐13	3.03E‐12
H19	2.65	0.82	3.23	1.26E‐03	4.13E‐03
SPP1	7.52	0.75	10.02	1.23E‐23	5.72E‐22
SERPINA3	9.08	0.96	9.43	4.19E‐21	1.55E‐19
CDH1	2.86	0.32	8.88	6.58E‐19	2.03E‐17
NEAT1	1.81	0.44	4.07	4.60E‐05	2.14E‐04
KRT8	1.35	0.26	5.17	2.32E‐07	1.69E‐06
CFTR	4.44	0.38	11.55	7.62E‐31	6.46E‐29
C3	7.81	0.95	8.22	2.03E‐16	4.89E‐15
ATP1B1	3.29	0.37	8.79	1.51E‐18	4.49E‐17
TNFAIP2	6.63	0.55	12.10	9.95E‐34	1.04E‐31
SPTBN1	1.96	0.28	6.94	3.99E‐12	5.67E‐11
ALDH1A3	4.83	0.57	8.44	3.22E‐17	8.35E‐16

^a^ DEseq2 normalized counts were filtered for those genes with a standard deviation of ≤1000 for all ALI samples (ND2.0 ALIw3, ND2.0 ALIw5, CWRU205 ALIw3 and CWRU205 ALIw5). Using the filtered list, genes are presented in order of high to low by subtracting the average DEseq2 normalized counts from all other stages from the average DEseq2 normalized counts for ALI samples.

^b^ Adjusted *P*‐value is calculated with the Benjamini‐Hochberg procedure.

To confirm the differentiation protocol was as expected based on previous publications,^9^, ^10^ we examined the expression of a number of known markers by both RT‐qPCR and RNA‐seq, including the key TFs SOX17 and FOXJ1. As expected, SOX17, an important marker of endoderm differentiation,^9^, ^10^ is >3‐fold higher in DE cells compared to AFE, and levels decline further as cells enter lung progenitor stages (Figure 3Ci). Expression of FOXJ1, which marks ciliated epithelial cells in the proximal airway,^61^ is maintained through ALIw5 (Figure 3Cii). Other markers for airway epithelial cells that are expressed through ALIw5 include the cell surface‐associated mucin MUC4, the gel‐forming mucin MUC5B, and the basal cell progenitor marker tumour protein p63 (TP63) (see GEO GSE136859, CompletePath_Counts.csv). Also shown in Figure 3D are immunofluorescence images for the lung and thyroid lineage marker NK2 homeobox 1 (NKX2.1), the FOXA2 transcription factor and the club cell marker secretoglobin family 1A member 1 (SCGBG1A1/CC10) in iPSC ND2.0‐derived ALIw5 cells. These data suggest that the cells are undergoing successful differentiation to the multiple cell types found within the proximal airway epithelium, though do not exclude the persistence of sub‐populations of non‐lung fated cells.

**Figure 4:**
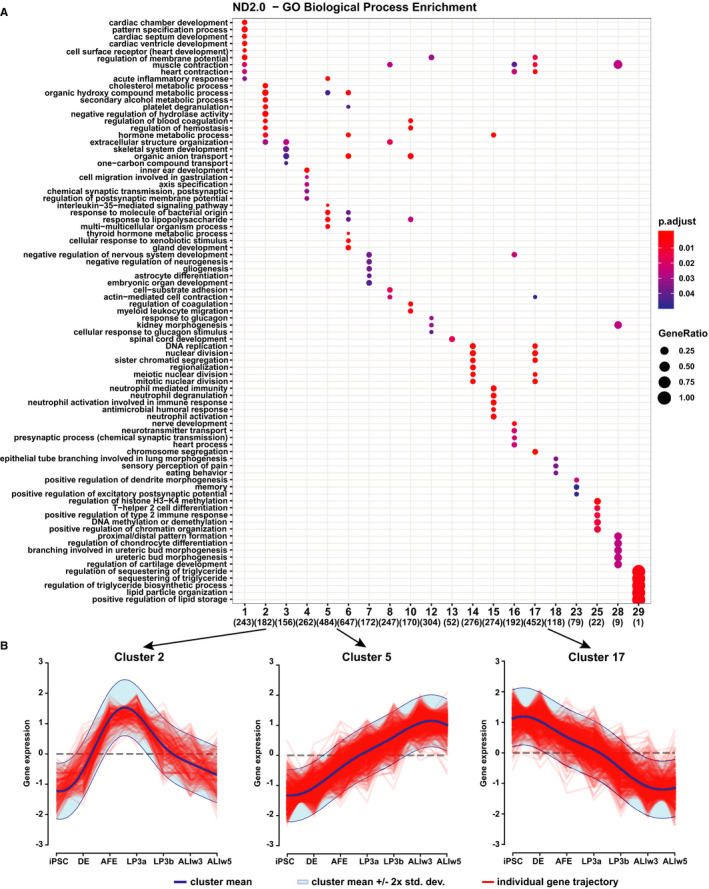
Gene ontology (GO) biological process enrichment analysis identifies key cellular, developmental and immune pathways. A. Dot plot of GO biological process terms enriched in gene expression clusters identified in the differentiation of ND2.0. Clustering was performed using an infinite Gaussian process mixture model. Only trajectories with statistically significant enrichment for a biological process category (20/29) are shown. B. Gene expression trajectories from iPSC to ALIw5 for Clusters 2, 5 and 17. The expression profile for individual genes across the developmental pathway (Table S2) is stratified into cluster models with the aggregate mean and standard deviation plotted for each

Next, to identify key biological processes that alter during the course of differentiation from DE to ALIw5 we performed clustering with an infinite Gaussian process mixture model.^62^ Data in Figure 4A show the GO biological process enrichment analysis with time in the ND2.0 iPSC line. Only 20 of the 29 clusters of genes with terms passing statistical significance (*P*‐value < 0.05) across a wide spectrum of biological functions are shown. Among processes of particular relevance to the lung epithelium are ‘epithelial tube branching involved in lung morphogenesis’ and several involved in immune and antimicrobial responses. Also of note are examples of the different gene expression profiles shown by individual gene clusters (Figure 4B and Figure S2). Cluster 2 genes show a peak of expression between AFE and LP3 with no transcripts at the start and end of the differentiation pipeline. Cluster 5 genes show no expression at the early stages of differentiation with a gradual increase through AFE and the LP stages, while reaching a plateau at ALI. The gene lists associated with each cluster are shown in Table S2. Among genes in Cluster 5 are some that are involved in innate immunity and the response to pathogens. The genes in Cluster 17 are highly expressed at the start of differentiation and are lost through differentiation, being silenced by ALIw3. Many similar pathways were enriched in clusters identified in the CWRU205 line, although the distributions are different (Figure S3 and S4).

### 3.3 Expression of genes involved in key pathways of lung development shows substantial changes between stages of differentiation

**Figure 5:**
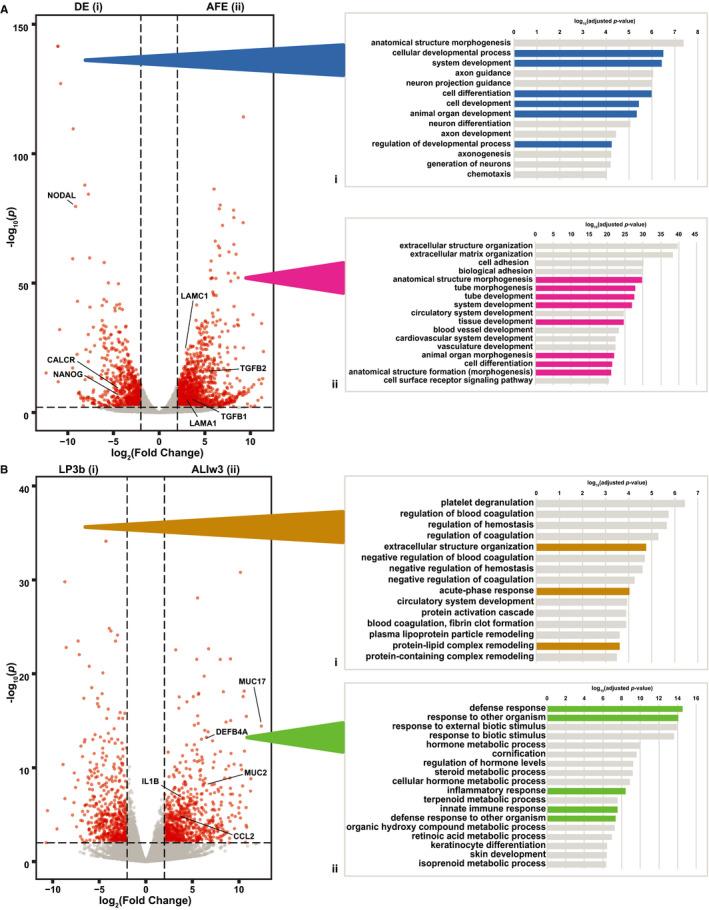
Pairwise transition gene ontology process enrichment analysis identifies key developmental and innate immune response processes. Volcano plot of weighted fold change as a function of *P*‐values for genes during the transitions from A) DE to AFE and B) LP3b to ALIw3. DEGs passing a ‐log_10_ adjusted *P*‐value of 2 and log_2_ fold change of 2 or −2 determined to be stage specific were processed using gene ontology process enrichment analysis. Biological process terms were chosen by passing a log_10_ adjusted *P*‐value with Bonferroni correction of 4 (Ai), 20 (Aii), 3 (Bi) or 6 (Bii). Notable categories are highlighted in colour for each analysis

To further delineate the biological functions that are significantly altered across individual stages of differentiation, we filtered the DEG data sets for each linear pairwise RNA‐seq comparison. Here, we focused on transitions between DE to AFE (Figure 5A) and LP3b to ALIw3 (Figure 5B) due to their relevance to early lung tissue development and airway epithelial cell identity, respectively. DEGs passing a 0.01 adjusted *P*‐value threshold were stratified by fold change of ±2 and then analysed using the PANTHER database (r5.1) and gProfiler (r5.2) to identify biological process enrichment gene ontology terms.

In the transition from DE to AFE, 633 genes were down‐regulated (Figure 5Ai) and 1107 genes were up‐regulated (Figure 5Aii), illustrating the occurrence of both substantial repression and activation of genes during this key developmental modification. A significant enrichment of genes associated with general pathways of cell and organ development was seen in DE cells (Figure 5Ai, in blue and Table S3). Among the DE‐specific DEGs are the NANOG homeobox gene (*NANOG*) and the Nodal Growth Differentiation Factor (*NODAL*), which both have pivotal roles in early development. Also overexpressed in DE cells are fibroblast growth factor 8 (*FGF8*), which has broad ranging functions in regulating embryonic development and cell proliferation, migration and differentiation, and Ripply 1 transcriptional repressor 1 (*RIPPLY1*), which has a critical role in somitogenesis. In contrast, genes more highly expressed in AFE cells are involved in anatomical structure development and, more specifically, tube development (Figure 5Aii, in pink and Table S4). Notably, a profound enrichment of genes involved in epithelial processes such extracellular matrix production and cell adhesion was evident in AFE‐specific DEGs. These include Transforming Growth Factor Beta 1 and 2 (*TGFB1, TGFB2*) and the Laminin Subunits Alpha 1, Alpha 2, Gamma 1 and Gamma 2 (*LAMA1*, *LAMA2*, *LAMC1*, *LAMC2*). These findings are consistent with the specialization of anterior foregut endoderm and loss of stem cell pluripotency.

Fewer genes showed significant alterations in expression levels between LP3b and ALIw3 (Figure 5B) where 344 are down‐regulated (Figure 5Bi) and 445 up‐regulated (Figure 5Bii). Genes up‐regulated in the transition from LP3b to ALIw3 were enriched for many processes integral to epithelial cell function (Figure 5Bii, green and Table S5). These include defence responses, including innate immunity, inflammatory response and several metabolic pathways. Among the ALIw3‐specific genes contributing to these processes are multiple cytokines such as C‐C motif Chemokines ligand 2 and 7 (*CCL2* and *CCL7*), interleukin 1 beta (*IL1B*), interleukin receptor like 1 (*IL1RL1*) and interleukin 1 receptor agonist (*IL1RN*), which inhibits IL1B activity, and the antimicrobial peptide defensin beta 4A (*DEFB4A*). Similarly, multiple membrane‐associated mucins including *MUC1*, *MUC3A*, *MUC13*, *MUC17* and *MUC21* are significantly up‐regulated between LP3b and ALIw3. Genes involved in these pathways are essential for the proper airway epithelial integrity and response to the environment. Many of the pathways up‐regulated in the differentiated ALI cultures are impaired by lung disease.

### 3.4 Intersecting open chromatin with differential gene expression through differentiation from DE to ALI cultures

**Figure 6:**
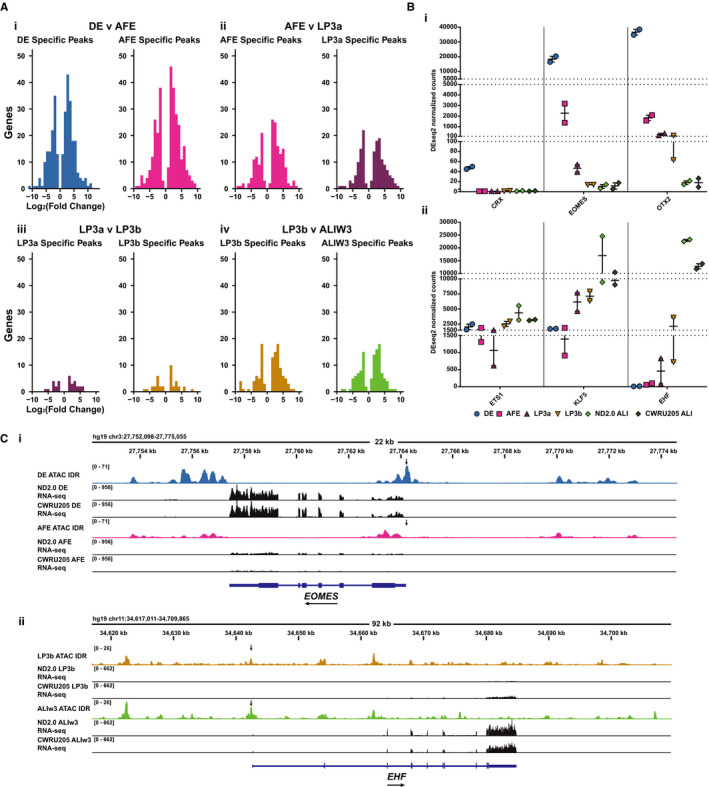
Differential gene expression correlates with presence of open chromatin. A, Distributions of stage‐specific DEGs correlated with ATAC‐seq peaks unique to either stage. Genes passing a 0.01 adjusted *P*‐value threshold with a peak within 20 kb of the gene body were binned by fold change for each linear pairwise comparison. Comparisons shown are i) DE to AFE, ii) AFE to LP3a, iii) LP3a to LP3b, iv) LP3b to ALIw3. B, RNA‐seq DEseq normalized counts for TFs with stage‐specific overrepresented motifs (by HOMER) in ATAC peaks i) DE‐specific TFs, CRX, EOMES and OTX2; ii) LP3b‐ALIw3/5 epithelial selective transcription factors, ETS1, KLF5 and EHF. C. Integrated Genomics Viewer (IGV) browser view of ATAC‐seq tracks and RNA‐seq read alignments at i) *EOMES* and ii) *EHF* loci comparing DE and AFE, or LP3b and ALIw3 stages, respectively. Arrows indicate stage‐dependent changes in chromatin accessibility at gene promoters

Next, we investigated the correlation between open chromatin within 20 kb of gene bodies and differential gene expression from the RNA‐seq data. DEG results for each linear transition through the differentiation were combined with annotation data for ATAC‐seq peaks specific to each gene. DEGs with at least one peak passing the IDR threshold for each stage were binned by log_2_ fold change in the pairwise RNA‐seq comparison (Figure 6A). While the overlap analysis shows consistent gene activation and repression around regions of open chromatin, substantially more genes are involved in the early DE to AFE transition (Figure 6Ai), somewhat lower but equivalent numbers are seen at the AFE to LP3a and LP3b to ALIw3 transitions (Figure 6Aii, iv), while many fewer are evident between LP3a and LP3b (Figure 6Aiii). These data are consistent with the major shift in cellular identity from DE to AFE and the more gradual changes through later differentiation and upon transition to ALI.

To reveal more about the TFs driving these changes in cellular identity and differentiation, we examined the expression profiles of specific TFs with important roles in early‐stage or late‐stage differentiation, for which binding motifs are enriched at peaks of open chromatin (Figure 6B). Among the top enriched motifs in DE cells, 12 are observed only in DE open chromatin (Figure 2C, Figure S1); moreover, 6 are also DE DEGs, including genes encoding the important differentiation factors EOMES and OTX2. Figure 6Bi shows the DEseq2 normalized counts at each state of differentiation for Cone‐Rod Homeobox (*CRX*), *EOMES* and *OTX2*. While *CRX* expression is only seen in DE cells, *EOMES* and *OTX2* show gradual reduction in activity through LP3b and ALI, respectively. Expression of *KLF5*, for which the binding motif is significantly enriched in peaks of open chromatin in AFE cells and all later stages, gradually increases through post‐AFE differentiation, in line with its role in airway epithelial cell differentiation (Figure 6Bii). In contrast to the abundance of ETS1, which is constant through differentiation, expression of *EHF,* for which the binding motif is only enriched in ALIw3 and ALIw5 cells, reaches maximal expression levels in ALI cells. This is consistent with previous reports of the role of EHF on lung epithelial function and modifying cystic fibrosis lung disease severity.^63^, ^64^, ^65^ To highlight the concordance between the ATAC‐seq signal and gene expression levels, we surveyed the open chromatin and RNA‐seq profiles at the *EOMES* and *EHF* loci. Both genes show some positive correlation of ATAC‐seq peak intensity and RNA‐seq reads (Figure 6C). Most notable are alterations in ATAC‐seq signal adjacent to the gene promoters that correspond to changes in gene expression level, evident from total aligned reads (Figure 6C). For example, loss of the ATAC‐seq signal at the *EOMES* promoter in the transition of DE to AFE correlates with reduced aligned RNA‐seq reads in AFE cells (Figure 6Ci). In contrast, gain of open chromatin at the *EHF* promoter correlates with increased aligned reads at the EHF locus in ALIw3 compared to LP3b (Figure 6Cii).

### 3.5 Comparison of iPSC‐derived ALI open chromatin and transcriptome with donor‐derived HBE‐ALI cultures

**Figure 7:**
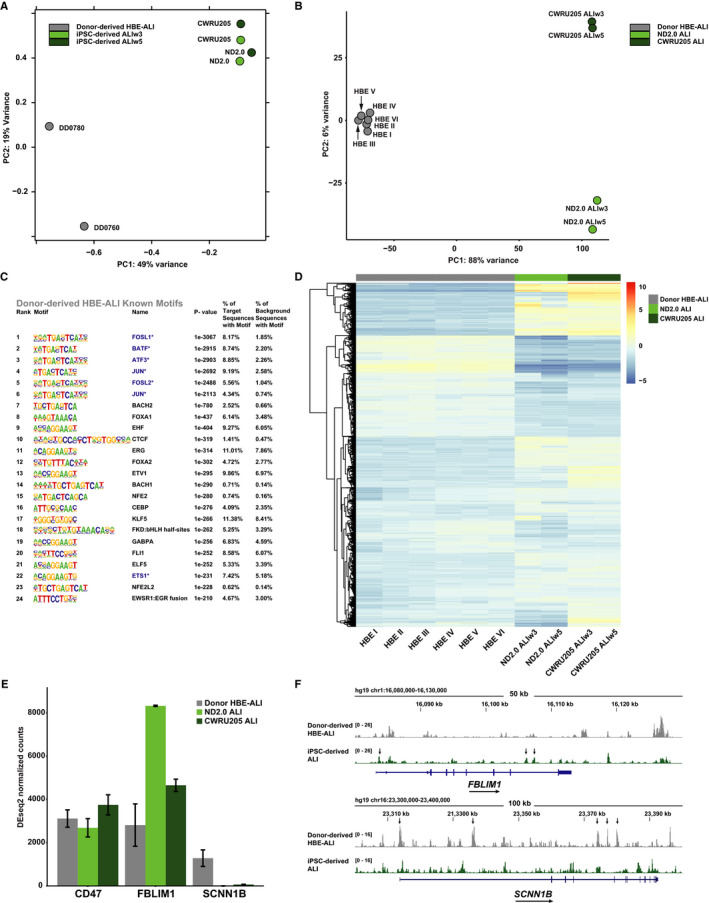
Comparison of ATAC‐seq and RNA‐seq profiles of donor‐derived ALI‐HBE and iPSC‐derived ALI‐HBE cultures. A, PCA plot for ATAC‐seq peak distribution comparing primary HBE‐ALI cultures from 2 donors (DD076O and DD078O) and 2 samples each from iPSC‐derived HBE‐ALI cultures CWRU205 and ND2.0. B, PCA plot for RNA‐seq gene expression data comparing HBE‐ALI cultures from 6 donors and 2 replicas each from ALI‐HBE ND2.0 and CWRU205. C, HOMER analysis showing the top 24 known TF binding motifs overrepresented in ATAC‐seq peaks from donor‐derived HBE‐ALI cultures. * and blue font denotes motifs also enriched in top 24 known motifs in iPSC‐derived ALIw3 cells (Figure 2Civ). D, Heatmap of the top 8645 DEGs comparing donor‐derived primary HBE‐ALI to iPSC‐derived ALI‐HBE samples passing a 0.01 adjusted *P*‐value threshold. Each donor for primary cells or iPSC‐derived cultures was used as a replicate for hierarchal clustering. E, Normalized sequence counts comparing donor‐derived and iPSC‐derived cells for *CD47* control and *FBLIM1* and *SCNN1B* DEGs. F, UCSC genome browser graphic of ATAC‐seq tracks (IDR) for donor‐derived and iPSC‐derived HBE‐ALI cultures at the *FBLM1* and *SCNN1B* loci. Arrows show sites of increased chromatin accessibility in cells with higher gene expression

A critical feature of the iPSC ALI cultures is how well they mirror the transcriptome and functions of ALI cultures generated from adult human bronchial epithelial cells derived from donor lung tissue. We observed significant differences in both open chromatin (Figure 7A) and gene expression profiles (Figure 7B) between the ALI cultures of different origin. The PCA plots of ATAC‐seq peaks show that while biological replicas of iPSC and donor‐derived ALI cultures cluster well by origin, they are far from each other, and there is more similarity between the two iPSC lines than the two donors (Figure 7A). With respect to gene expression profiles (Figure 7B), the six replicas of donor‐derived HBE‐ALI cells cluster well. Though the 2 iPSC‐derived lines are quite different from the donor‐derived HBE‐ALI cells there is only a 6% variance between the biological replicates. Technical replicates of each line also cluster well, reflecting robust and consistent culture protocols. Despite the divergence between iPSC‐ and donor‐derived ALI cultures, the top 24 known TF binding motifs overrepresented in the IDR open chromatin peaks of HBE‐ALI cells (Figure 7C) show some overlap with iPSC‐derived ALI cells (Figure 2Civ and Figure S1C). Key factors regulating normal airway epithelial function including EHF, KLF5, and BTB domain and CNC homolog 2 (BACH2) are among these TFs in common. Unique to donor‐derived ALI cells are overrepresented motifs for CCAAT enhancer binding protein (CEBP), BACH1 and E47‐like ETS transcription factor 5 (ELF5). The matrix of differential gene expression (Figure 7D) also shows significant differences between the HBE‐ALI cultures of different origins. A total of 3394 genes had a ≥2‐fold higher expression in donor‐derived HBE‐ALI, and 3478 genes were more highly expressed in the iPSC‐derived ALI cells. A gene ontology process enrichment analysis on these DEGs (Table S6) showed that processes related to cilia and microtubules were the most markedly up‐regulated in donor‐derived HBE cultures, consistent with failure of the iPSC‐derived cells to generate a robust ciliated cell compartment. In contrast, iPSC‐derived ALI cultures showed an increase in processes associated with cell surface components, extracellular matrix and cell adhesion. The DEG matrix also illustrates the high degree variability between the two iPSC‐derived ALI cultures consistent with data shown in Figure 3A and B. We then examined the relationship between open chromatin profiles and gene expression levels for two genes, Filamin‐binding LIM Protein 1 (*FBLIM1*), which is more highly expressed in iPSC‐derived ALI cultures, and Sodium Channel Epithelial 1 Beta Subunit (*SCNN1B*) (encoding the beta subunit of ENaC), which is more abundant in donor‐derived HBE‐ALI cells (Figure 7E). Sites of open chromatin at each locus correlated well with differences in gene expression, with more pronounced accessibility at the promoters, introns and downstream elements in the cultures showing higher expression (Figure 7F). CD47 provided a negative control for this analysis, as neither the normalized counts from DESeq2 nor the ATAC‐seq profiles (Figure 7E and Figure S5) vary between the cultures.

## 4 DISCUSSION

The goal of the experiments described here was to generate unbiased genome‐wide data on open chromatin and gene expression during the differentiation of iPSCs into lung epithelial cells at ALI, as this model is frequently used for the study of key events in lung disease such as CF. Though careful characterization of the expression of multiple specific marker genes along the differentiation pathway has been performed by several groups,^7^, ^8^, ^9^, ^10^, ^11^, ^12^, ^13^, ^14^, ^18^, ^20^, ^21^, ^22^, ^23^ these studies of individual transcripts or their encoded proteins are by definition limited in scope. The definition of key markers has generally relied on extensive data from other mammalian species, most notably the mouse, which is exceptionally well studied (reviewed in^66^). However, the mouse lung has a number of important anatomical and functional differences from the human lung,^67^, ^68^ suggesting that an unbiased review of gene expression profiles in the iPSC‐to‐ALI differentiation could be valuable. Hence, the genome‐wide data reported here are important contributions to understanding the functional genomics of this differentiation pipeline. Several aspects of the differentiation warrant further discussion; although iPSC‐derived differentiated cellular models are widely used in medical research, and have facilitated numerous important advances, some pathways of differentiation are more robust than others. Indeed, there are ongoing efforts to optimize the protocols for generating well‐differentiated bronchial epithelial cells at ALI from iPSCs. At the time of these experiments, we followed one well‐documented protocol of this differentiation pipeline (Figure 1)^10^ but are aware that more recent optimizations may enhance the outcome.^11^, ^13^, ^21^, ^22^ Two key questions warrant further discussion: first, how robust is the differentiation pipeline in different iPSC lines and second, how similar are iPSC‐derived HBE‐ALI cultures to organ donor‐derived HBE‐ALI cells.

To address the first question, we investigated the reproducibility of differentiation in terms of variable efficiency and cell heterogeneity in three iPSC cell lines, from two sources. One line repeatedly failed to reproducibly differentiate to the ALI stage and was excluded for further experimentation and analysis. While the other two lines reproducibly differentiated to ALI cultures, one line apparently differentiated much more efficiently than the other. This phenomenon is well illustrated by the divergence between the ALIw3 and ALIw5 transcription profiles of the two donor lines. After 5 weeks of ALI culture, each line was more similar to itself after 3 weeks than to the other culture at 5 weeks. Though ND2.0 ALI cells expressed many markers for proximal lung progenitors (*TP63* and *KRT5* (basal cells), *FOXJ1* (ciliated cells), *ARG2* (goblet cells)), others were expressed at lower levels compared to CWRU205 ALI cells. The latter appeared to provide a more robust model airway epithelium at ALI including expressing markers for secretory cells (*SCGB3A2*) and goblet cells (*MUC5AC*), in addition to the cell types seen in ND2.0 ALI cultures. In the context of the divergent differentiation of the CWRU205 and ND2.0 cultures, it is relevant that differentiation of definitive endoderm into airway cells is often associated with the production of non‐lung endoderm‐derived cells types. These include predominately hepatic‐like cells in iPSC‐derived proximal airway,^9^, ^10^, ^18^, ^19^, ^20^ gastric‐like cells in iPSC‐derived distal airway^20^ and other endoderm‐derived tissue lineages such as pancreas.^9^, ^10^ The differentiated ALI cultures we generated from iPSC cells similarly expressed some markers for non‐lung endoderm cells. These include the hepatic markers fibrinogen beta chain (*FGB*), apolipoprotein A2 (*APOA2*) and hepatocyte nuclear factor 4A (*HNF4A*) and to a lesser extent, pancreas as shown by pancreatic and duodenal homeobox (*PDX1*) expression. The different abundance of non‐lung cell types likely contributes to the deviation of expression and open chromatin profiles between the two iPSC‐derived ALI cultures.

Directly addressing the second question, there are clear differences between open chromatin and expression profiles of human donor‐derived HBE‐ALI and ALI derived from iPSCs (Figure 7). Many transcription factors that are expressed in murine lung epithelial cells, and play a role in their differentiation, are highly expressed in both groups of ALI cells in addition to enrichment of their binding motifs in both cell types (Figure 2Civ, Figure S1C, Figure 7C). These TFs include JUN, KLF5 and EHF. However, the binding motifs of other TFs with an important role in the lung epithelium are not enriched in open chromatin from iPSC‐derived ALI, despite being similarly expressed in iPSC‐ and donor‐derived ALI cultures. These factors include members of the CEBP family,^69^ BACH1,^70^ NFE2L2 (formerly NRF2) ^71^ and ELF5.^72^ The fact that these TFs are present in the cells but are apparently not being selectively recruited to *cis*‐regulatory elements (open chromatin peaks) suggests that critical co‐factors required for DNA‐binding might be absent or mis‐expressed. Alternatively, these TFs may not be appropriately activated by external stimuli in the iPSC‐derived ALI cultures, including from other cell types, and so cannot bind DNA. It is also likely that some of the cell types in the donor‐derived HBE‐ALI cultures that contribute to the motif enrichment signal are either absent or at lower abundance in the iPSC‐derived ALI cells.

In conclusion, we provide a detailed molecular characterization of the gene expression repertoire and the activating and repressive TFs that direct the differentiation of iPSCs into lung epithelial cells at ALI. Our data show that though this model to study lung epithelial function in health and disease has many advantages, according to the protocols we used it currently falls short of producing a fully differentiated functional airway epithelium. This may reflect contamination with non‐lung fated cells together with a lack of appropriate environmental cues, which may be biochemical (such as appropriate growth factors) or physical (lack of suitable matrix or substrate and stretch forces^73^), which could result in failure to produce rare but critical cell types. Further higher resolution molecular analysis by single cell RNA‐seq and single cell ATAC‐seq may reveal the absent components. The application of our analysis pipeline to other protocols for the differentiation of iPSC into lung epithelial cells^13^, ^14^, ^19^, ^21^, ^22^, ^23^, ^74^ would likely be highly informative.

## CONFLICTS OF INTEREST

The authors confirm that there are no conflicts of interest. The funders had no role in the design of the study, in the collection, analyses or interpretation of the data, in the writing of the manuscript, or in the decision to publish the results.

## AUTHOR CONTRIBUTION

**Jenny L Kerschner:** Conceptualization (supporting); Data curation (equal); Formal analysis (equal); Investigation (equal); Methodology (equal); Writing‐original draft (supporting); Writing‐review & editing (supporting). **Alekh Paranjapye:** Conceptualization (supporting); Data curation (equal); Formal analysis (equal); Investigation (equal); Methodology (equal); Writing‐original draft (supporting); Writing‐review & editing (supporting). **Shiyi Yin:** Data curation (supporting); Formal analysis (supporting); Investigation (supporting); Methodology (supporting). **Dannielle L Skander:** Data curation (supporting); Formal analysis (supporting); Investigation (supporting); Methodology (supporting). **Gurkan Bebek:** Data curation (supporting); Formal analysis (equal); Investigation (supporting); Methodology (supporting); Software (equal). **Shih‐Hsing Leir:** Conceptualization (supporting); Data curation (supporting); Formal analysis (supporting); Investigation (supporting); Methodology (equal). **Ann Harris:** Conceptualization (lead); Data curation (equal); Formal analysis (equal); Funding acquisition (lead); Investigation (supporting); Methodology (supporting); Project administration (lead); Writing‐original draft (lead); Writing‐review & editing (lead).

## Supporting information

Supplementary Material

## Data Availability

Genome‐wide data are deposited at GEO:GSE136859.

## References

[1] Takahashi K , Tanabe K , Ohnuki M , et al. Induction of pluripotent stem cells from adult human fibroblasts by defined factors. Cell. 2007;131:861‐872. 18035408 10.1016/j.cell.2007.11.019

[2] Yu J , Vodyanik MA , Smuga‐Otto K , et al. Induced pluripotent stem cell lines derived from human somatic cells. Science. 2007;318:1917‐1920. 18029452 10.1126/science.1151526

[3] Rowe RG , Daley GQ . Induced pluripotent stem cells in disease modelling and drug discovery. Nat Rev Genet. 2019;20:377‐388. 30737492 10.1038/s41576-019-0100-z PMC6584039

[4] Whitcutt MJ , Adler KB , Wu R . A biphasic chamber system for maintaining polarity of differentiation of cultured respiratory tract epithelial cells. Vitro Cell Dev Biol. 1988;24:420‐428. 10.1007/BF02628493 3372447

[5] Fulcher ML , Gabriel S , Burns KA , Yankaskas JR , Randell SH . Well‐differentiated human airway epithelial cell cultures. Methods Mol Med. 2005;107:183‐206. 15492373 10.1385/1-59259-861-7:183

[6] Randell SH , Fulcher ML , O'Neal W , Olsen JC . Primary epithelial cell models for cystic fibrosis research. Methods Mol Biol. 2011;742:285‐310. 21547740 10.1007/978-1-61779-120-8_18

[7] Somers A , Jean JC , Sommer CA , et al. Generation of transgene‐free lung disease‐specific human induced pluripotent stem cells using a single excisable lentiviral stem cell cassette. Stem Cells. 2010;28:1728‐1740. 20715179 10.1002/stem.495 PMC3422663

[8] Ikonomou L , Kotton DN . Derivation of endodermal progenitors from pluripotent stem cells. J Cell Physiol. 2015;230:246‐258. 25160562 10.1002/jcp.24771 PMC4344429

[9] Wong AP , Bear CE , Chin S , et al. Directed differentiation of human pluripotent stem cells into mature airway epithelia expressing functional CFTR protein. Nature Biotechnol. 2012;30:876‐882. 22922672 10.1038/nbt.2328 PMC3994104

[10] Wong AP , Chin S , Xia S , Garner J , Bear CE , Rossant J . Efficient generation of functional CFTR‐expressing airway epithelial cells from human pluripotent stem cells. Nat Protoc. 2015;10:363‐381. 25654755 10.1038/nprot.2015.021

[11] Firth AL , Dargitz CT , Qualls SJ , et al. Generation of multiciliated cells in functional airway epithelia from human induced pluripotent stem cells. Proc Natl Acad Sci USA. 2014;111:E1723‐E1730. 24706852 10.1073/pnas.1403470111 PMC4035971

[12] Gomperts BN . Induction of multiciliated cells from induced pluripotent stem cells. Proc Natl Acad Sci U S A. 2014;111:6120‐6121. 24740182 10.1073/pnas.1404414111 PMC4035920

[13] Huang SX , Green MD , de Carvalho AT , et al. The in vitro generation of lung and airway progenitor cells from human pluripotent stem cells. Nat Protoc. 2015;10:413‐425. 25654758 10.1038/nprot.2015.023 PMC4654940

[14] Huang SX , Islam MN , O'Neill J , et al. Efficient generation of lung and airway epithelial cells from human pluripotent stem cells. Nat Biotechnol. 2014;32:84‐91. 24291815 10.1038/nbt.2754 PMC4101921

[15] Konishi S , Gotoh S , Tateishi K , et al. Directed induction of functional multi‐ciliated cells in proximal airway epithelial spheroids from human pluripotent stem cells. Stem Cell Reports. 2016;6:18‐25. 26724905 10.1016/j.stemcr.2015.11.010 PMC4720023

[16] Miller AJ , Dye BR , Ferrer‐Torres D , et al. Generation of lung organoids from human pluripotent stem cells in vitro. Nat Protoc. 2019;14:518‐540. 30664680 10.1038/s41596-018-0104-8 PMC6531049

[17] Crane AM , Kramer P , Bui JH , et al. Targeted correction and restored function of the CFTR gene in cystic fibrosis induced pluripotent stem cells. Stem Cell Reports. 2015;4:569‐577. 25772471 10.1016/j.stemcr.2015.02.005 PMC4400651

[18] Hawkins F , Kramer P , Jacob A , et al. Prospective isolation of NKX2‐1‐expressing human lung progenitors derived from pluripotent stem cells. J Clin Invest. 2017;127:2277‐2294. 28463226 10.1172/JCI89950 PMC5451263

[19] McCauley KB , Hawkins F , Serra M , Thomas DC , Jacob A , Kotton DN . Efficient derivation of functional human airway epithelium from pluripotent stem cells via temporal regulation of Wnt signaling. Cell Stem Cell. 2017;20:844‐857.e6. 28366587 10.1016/j.stem.2017.03.001 PMC5457392

[20] McCauley KB , Alysandratos KD , Jacob A , et al. Single‐cell transcriptomic profiling of pluripotent stem cell‐derived SCGB3A2+ airway epithelium. Stem Cell Reports. 2018;10:1579‐1595. 29657097 10.1016/j.stemcr.2018.03.013 PMC5995784

[21] Jacob A , Morley M , Hawkins F , et al. Differentiation of human pluripotent stem cells into functional lung alveolar epithelial cells. Cell Stem Cell. 2017;21:472‐488.e10. 28965766 10.1016/j.stem.2017.08.014 PMC5755620

[22] Rankin SA , Han L , McCracken KW , et al. A retinoic acid‐hedgehog cascade coordinates mesoderm‐inducing signals and endoderm competence during lung specification. Cell Rep. 2016;16:66‐78. 27320915 10.1016/j.celrep.2016.05.060 PMC5314425

[23] Rankin SA , McCracken KW , Luedeke DM , et al. Timing is everything: Reiterative Wnt, BMP and RA signaling regulate developmental competence during endoderm organogenesis. Dev Biol. 2018;434:121‐132. 29217200 10.1016/j.ydbio.2017.11.018 PMC5785443

[24] Rankin SA , Zorn AM . Gene regulatory networks governing lung specification. J Cell Biochem. 2014;115:1343‐1350. 24644080 10.1002/jcb.24810 PMC4263286

[25] Schuger L , Skubitz AP , Gilbride K , Mandel R , He L . Laminin and heparan sulfate proteoglycan mediate epithelial cell polarization in organotypic cultures of embryonic lung cells: evidence implicating involvement of the inner globular region of laminin beta 1 chain and the heparan sulfate groups of heparan sulfate proteoglycan. Dev Biol. 1996;179:264‐273. 8873769 10.1006/dbio.1996.0256

[26] Kalluri R . Basement membranes: structure, assembly and role in tumour angiogenesis. Nat Rev Cancer. 2003;3:422‐433. 12778132 10.1038/nrc1094

[27] Ghaedi M , Calle EA , Mendez JJ , et al. Human iPS cell‐derived alveolar epithelium repopulates lung extracellular matrix. J Clin Invest. 2013;123:4950‐4962. 24135142 10.1172/JCI68793 PMC3809786

[28] Buenrostro JD , Wu B , Chang HY , Greenleaf WJ . ATAC‐seq: A method for assaying chromatin accessibility genome‐wide. Curr Protoc Stem Cell Biol. 2015; 109: 1‐9. 10.1002/0471142727.mb2129s109 PMC4374986 25559105

[29] Yang R , Browne JA , Eggener SE , Leir S‐H , Harris A . A novel transcriptional network for the Androgen Receptor in human epididymis epithelial cells. Mol Hum Reprod. 2018;24:433‐443. 30016502 10.1093/molehr/gay029 PMC6454485

[30] Browne JA , Yang R , Eggener SE , Leir SH , Harris A . HNF1 regulates critical processes in the human epididymis epithelium. Mol Cell Endocrinol. 2016;425:94‐102. 26808453 10.1016/j.mce.2016.01.021 PMC4799753

[31] Wang Q , Gu L , Adey A , et al. Tagmentation‐based whole‐genome bisulfite sequencing. Nat Protoc. 2013;8:2022‐2032. 24071908 10.1038/nprot.2013.118

[32] Dobin A , Davis CA , Schlesinger F , et al. STAR: ultrafast universal RNA‐seq aligner. Bioinformatics. 2013;29:15‐21. 23104886 10.1093/bioinformatics/bts635 PMC3530905

[33] Liao Y , Smyth GK , Shi W . featureCounts: an efficient general purpose program for assigning sequence reads to genomic features. Bioinformatics. 2014;30:923‐930. 24227677 10.1093/bioinformatics/btt656

[34] Love MI , Huber W , Anders S . Moderated estimation of fold change and dispersion for RNA‐seq data with DESeq2. Genome Biol. 2014;15:550. 25516281 10.1186/s13059-014-0550-8 PMC4302049

[35] Goodale BC , Rayack EJ , Stanton BA . Arsenic alters transcriptional responses to Pseudomonas aeruginosa infection and decreases antimicrobial defense of human airway epithelial cells. Toxicol Appl Pharmacol. 2017;331:154‐163. 28625800 10.1016/j.taap.2017.06.010 PMC5568502

[36] Mi H , Muruganujan A , Ebert D , Huang X , Thomas PD . PANTHER version 14: more genomes, a new PANTHER GO‐slim and improvements in enrichment analysis tools. Nucleic Acids Res. 2019;47:D419‐D426. 30407594 10.1093/nar/gky1038 PMC6323939

[37] Heinz S , Benner C , Spann N , et al. Simple combinations of lineage‐determining transcription factors prime cis‐regulatory elements required for macrophage and B cell identities. Mol Cell. 2010;38:576‐589. 20513432 10.1016/j.molcel.2010.05.004 PMC2898526

[38] Ross‐Innes CS , Stark R , Teschendorff AE , et al. Differential oestrogen receptor binding is associated with clinical outcome in breast cancer. Nature. 2012;481:389‐393. 22217937 10.1038/nature10730 PMC3272464

[39] Trapnell C , Roberts A , Goff L , et al. Differential gene and transcript expression analysis of RNA‐seq experiments with TopHat and Cufflinks. Nat Protoc. 2012;7:562‐578. 22383036 10.1038/nprot.2012.016 PMC3334321

[40] Wang S , Sun H , Ma J , et al. Target analysis by integration of transcriptome and ChIP‐seq data with BETA. Nat Protoc. 2013;8:2502‐2515. 24263090 10.1038/nprot.2013.150 PMC4135175

[41] Bell AC , West AG , Felsenfeld G . The protein CTCF is required for the enhancer blocking activity of vertebrate insulators. Cell. 1999;98:387‐396. 10458613 10.1016/s0092-8674(00)81967-4

[42] Klenova EM , Morse HC 3rd , Ohlsson R , Lobanenkov VV . The novel BORIS + CTCF gene family is uniquely involved in the epigenetics of normal biology and cancer. Semin Cancer Biol. 2002;12:399‐414. 12191639 10.1016/s1044-579x(02)00060-3

[43] Lentjes MH , Niessen HE , Akiyama Y , de Bruine AP , Melotte V , van Engeland M . The emerging role of GATA transcription factors in development and disease. Expert Rev Mol Med. 2016;18:e3. 26953528 10.1017/erm.2016.2 PMC4836206

[44] Tremblay M , Sanchez‐Ferras O , Bouchard M . GATA transcription factors in development and disease. Development. 2018;145. 10.1242/dev.164384 30348673

[45] Zaret KS , Carroll JS . Pioneer transcription factors: establishing competence for gene expression. Genes Dev. 2011;25:2227‐2241. 22056668 10.1101/gad.176826.111 PMC3219227

[46] Cirillo LA , Lin FR , Cuesta I , Friedman D , Jarnik M , Zaret KS . Opening of compacted chromatin by early developmental transcription factors HNF3 (FoxA) and GATA‐4. Mol Cell. 2002;9:279‐289. 11864602 10.1016/s1097-2765(02)00459-8

[47] Kaestner KH . The FoxA factors in organogenesis and differentiation. Curr Opin Genet Dev. 2010;20:527‐532. 20591647 10.1016/j.gde.2010.06.005 PMC2943037

[48] Tsankov AM , Gu H , Akopian V , et al. Transcription factor binding dynamics during human ES cell differentiation. Nature. 2015;518:344‐349. 25693565 10.1038/nature14233 PMC4499331

[49] Perea‐Gomez A , Lawson KA , Rhinn M , et al. Otx2 is required for visceral endoderm movement and for the restriction of posterior signals in the epiblast of the mouse embryo. Development. 2001;128:753‐765. 11171400 10.1242/dev.128.5.753

[50] Teo AK , Arnold SJ , Trotter MW , et al. Pluripotency factors regulate definitive endoderm specification through eomesodermin. Genes Dev. 2011;25:238‐250. 21245162 10.1101/gad.607311 PMC3034899

[51] She ZY , Yang WX . SOX family transcription factors involved in diverse cellular events during development. Eur J Cell Biol. 2015;94:547‐563. 26340821 10.1016/j.ejcb.2015.08.002

[52] Wan H , Luo F , Wert SE , et al. Kruppel‐like factor 5 is required for perinatal lung morphogenesis and function. Development. 2008;135:2563‐2572. 18599506 10.1242/dev.021964 PMC4459582

[53] Becker T , Loch G , Beyer M , et al. FOXO‐dependent regulation of innate immune homeostasis. Nature. 2010;463:369‐373. 20090753 10.1038/nature08698

[54] Seiler F , Hellberg J , Lepper PM , et al. FOXO transcription factors regulate innate immune mechanisms in respiratory epithelial cells. J Immunol. 2013;190:1603‐1613. 23315071 10.4049/jimmunol.1200596

[55] Wilkinson DG , Bhatt S , Ryseck RP , Bravo R . Tissue‐specific expression of c‐jun and junB during organogenesis in the mouse. Development. 1989;106:465‐471. 2480878 10.1242/dev.106.3.465

[56] Wan H , Dingle S , Xu Y , et al. Compensatory roles of Foxa1 and Foxa2 during lung morphogenesis. J Biol Chem. 2005;280:13809‐13816. 15668254 10.1074/jbc.M414122200

[57] Belo JA , Bachiller D , Agius E , et al. Cerberus‐like is a secreted BMP and nodal antagonist not essential for mouse development. Genesis. 2000;26:265‐270. 10748465

[58] Iwashita H , Shiraki N , Sakano D , et al. Secreted cerberus1 as a marker for quantification of definitive endoderm differentiation of the pluripotent stem cells. PLoS One. 2013;8:e64291. 23717584 10.1371/journal.pone.0064291 PMC3661443

[59] Meno C , Shimono A , Saijoh Y , et al. lefty‐1 is required for left‐right determination as a regulator of lefty‐2 and nodal. Cell. 1998;94:287‐297. 9708731 10.1016/s0092-8674(00)81472-5

[60] Borel F , Sun H , Zieger M , et al. Editing out five Serpina1 paralogs to create a mouse model of genetic emphysema. Proc Natl Acad Sci U S A. 2018;115:2788‐2793. 29453277 10.1073/pnas.1713689115 PMC5856518

[61] You Y , Huang T , Richer EJ , et al. Role of f‐box factor foxj1 in differentiation of ciliated airway epithelial cells. Am J Physiol Lung Cell Mol Physiol. 2004;286:L650‐L657. 12818891 10.1152/ajplung.00170.2003

[62] McDowell IC , Manandhar D , Vockley CM , Schmid AK , Reddy TE , Engelhardt BE . Clustering gene expression time series data using an infinite Gaussian process mixture model. PLoS Comput Biol. 2018;14:e1005896. 29337990 10.1371/journal.pcbi.1005896 PMC5786324

[63] Fossum SL , Mutolo MJ , Tugores A , et al. Ets homologous factor (EHF) has critical roles in epithelial dysfunction in airway disease. J Biol Chem. 2017;292:10938‐10949. 28461336 10.1074/jbc.M117.775304 PMC5491778

[64] Fossum SL , Mutolo MJ , Yang R , et al. Ets homologous factor regulates pathways controlling response to injury in airway epithelial cells. Nucleic Acids Res. 2014;42:13588‐13598. 25414352 10.1093/nar/gku1146 PMC4267623

[65] Stolzenburg LR , Yang R , Kerschner JL , et al. Regulatory dynamics of 11p13 suggest a role for EHF in modifying CF lung disease severity. Nucleic Acids Res. 2017;45:8773‐8784. 28549169 10.1093/nar/gkx482 PMC5587731

[66] Maeda Y , Dave V , Whitsett JA . Transcriptional control of lung morphogenesis. Physiol Rev. 2007;87:219‐244. 17237346 10.1152/physrev.00028.2006

[67] Borthwick DW , West JD , Keighren MA , Flockhart JH , Innes BA , Dorin JR . Murine submucosal glands are clonally derived and show a cystic fibrosis gene‐dependent distribution pattern. Am J Respir Cell Mol Biol. 1999;20:1181‐1189. 10340937 10.1165/ajrcmb.20.6.3475

[68] Shah VS , Meyerholz DK , Tang XX , et al. Airway acidification initiates host defense abnormalities in cystic fibrosis mice. Science. 2016;351:503‐507. 26823428 10.1126/science.aad5589 PMC4852973

[69] Flodby P , Barlow C , Kylefjord H , Ahrlund‐Richter L , Xanthopoulos KG . Increased hepatic cell proliferation and lung abnormalities in mice deficient in CCAAT/enhancer binding protein alpha. J Biol Chem. 1996;271:24753‐24760. 8798745 10.1074/jbc.271.40.24753

[70] Ebina‐Shibuya R , Watanabe‐Matsui M , Matsumoto M , et al. The double knockout of Bach1 and Bach2 in mice reveals shared compensatory mechanisms in regulating alveolar macrophage function and lung surfactant homeostasis. J Biochem. 2016;160:333‐344. 27387751 10.1093/jb/mvw041

[71] Chan K , Kan YW . Nrf2 is essential for protection against acute pulmonary injury in mice. Proc Natl Acad Sci U S A. 1999;96:12731‐12736. 10535991 10.1073/pnas.96.22.12731 PMC23072

[72] Metzger DE , Xu Y , Shannon JM . Elf5 is an epithelium‐specific, fibroblast growth factor–sensitive transcription factor in the embryonic lung. Dev Dyn. 2007;236:1175‐1192. 17394208 10.1002/dvdy.21133

[73] Nikolic MZ , Sun D , Rawlins EL . Human lung development: recent progress and new challenges. Development. 2018;145(16):dev163485. 30111617 10.1242/dev.163485 PMC6124546

[74] McCauley KB , Hawkins F , Kotton DN . Derivation of Epithelial‐Only Airway Organoids from Human Pluripotent Stem Cells. Curr Protoc Stem Cell Biol. 2018;45:e51. 30040246 10.1002/cpsc.51 PMC6060639

