# Supplementary material for: A functional genomics approach to investigate the differentiation of iPSCs into lung epithelium at air‐liquid interface
Source: J Cell Mol Med. 2020 Jul 21;24(17):9853–70. doi: 10.1111/jcmm.15568 (PMC7520342; doi:10.1111/jcmm.15568)
Supplement: Supplementary file 1 — Supplementary Material [file JCMM-24-9853-s001.pdf]

A iPSC Known Motifs

Rank	Motif	Name	P-value	% of Target Sequences with Motif	% of Background Sequences with Motif
1		CTCF	1e-312	1.89%	0.60%
2		CTCFL	1e-159	3.06%	1.71%
3		SP1	1e-62	5.26%	4.05%
4		NFY	1e-54	4.21%	3.20%
5		TEAD2	1e-47	2.24%	1.57%
6		TEAD	1e-46	2.52%	1.81%
7		OCT4/SOX2/ TCF(HNF4)/ NANOG sites	1e-46	0.54%	0.25%
8		ATF3	1e-43	2.24%	1.59%
9		OCT4	1e-41	1.29%	0.82%
10		FOSL1	1e-38	1.92%	1.35%
11		TEAD4	1e-38	3.74%	2.94%
12		JUN	1e-37	2.51%	1.87%
13		BATF	1e-37	2.17%	1.57%
14		KLF5	1e-37	13.86%	12.35%
15		SOX10	1e-36	5.93%	4.94%
16		FOSL2	1e-35	1.29%	0.86%
17		SOX3	1e-34	6.13%	5.16%
18		JUN	1e-31	0.96%	0.61%
19		SOX2	1e-30	3.10%	2.46%
20		FLI1	1e-29	7.92%	6.88%
21		SOX4	1e-29	3.13%	2.48%
22		ELK1	1e-29	5.29%	4.45%
23		OCT2	1e-28	0.81%	0.51%
24		BACH2	1e-25	0.79%	0.50%
25		ETV1	1e-24	8.49%	7.51%

B LP3a Known Motifs

Rank	Motif	Name	P-value	% of Target Sequences with Motif	% of Background Sequences with Motif
1		FOXA1	1e-154	3.02%	1.71%
2		FOXA1	1e-141	3.65%	2.25%
3		CTCF	1e-123	1.38%	0.63%
4		Forkhead: bHLH half-sites	1e-97	3.48%	2.31%
5		FOXA2	1e-91	2.61%	1.65%
6		SP1	1e-67	5.39%	4.14%
7		NFY	1e-65	4.30%	3.21%
8		CTCFL	1e-58	2.56%	1.78%
9		ELK4	1e-54	5.70%	4.54%
10		YY1	1e-46	0.85%	0.47%
11		KLF5	1e-42	14.16%	12.57%
12		ELK1	1e-40	5.51%	4.52%
13		JUN	1e-38	2.37%	1.75%
14		TEAD	1e-37	2.35%	1.74%
15		FOXP1	1e-37	1.30%	0.86%
16		GATA2	1e-35	1.90%	1.37%
17		TEAD2	1e-35	2.05%	1.50%
18		FOSL1	1e-34	1.74%	1.24%
19		SOX3	1e-33	6.03%	5.09%
20		TEAD4	1e-33	3.51%	2.80%
21		BATF	1e-32	1.99%	1.47%
22		JUN	1e-31	0.90%	0.57%
23		MAZ	1e-30	16.59%	15.15%
24		GATA4	1e-30	2.84%	2.23%
25		BACH2	1e-30	0.82%	0.52%

C ALIw5 Known Motifs

Rank	Motif	Name	P-value	% of Target Sequences with Motif	% of Background Sequences with Motif
1		CTCF	1e-125	1.64%	0.61%
2		SP1	1e-99	6.31%	4.23%
3		JUN	1e-90	1.39%	0.57%
4		NFY	1e-88	5.00%	3.26%
5		FOSL2	1e-77	1.70%	0.82%
6		ATF3	1e-69	2.66%	1.57%
7		FOSL1	1e-66	2.35%	1.35%
8		BATF	1e-65	2.67%	1.60%
9		CTCFL	1e-58	2.71%	1.68%
10		JUN	1e-53	2.89%	1.86%
11		KLF5	1e-49	14.71%	12.43%
12		ELK4	1e-42	6.43%	5.01%
13		BACH2	1e-41	1.07%	0.55%
14		YY1	1e-38	1.03%	0.54%
15		GFY:ZNF143 half-sites	1e-34	0.82%	0.41%
16		ETS	1e-34	3.63%	2.68%
17		FLI1	1e-31	8.91%	7.46%
18		ELK1	1e-30	6.24%	5.05%
19		ELF1	1e-28	5.53%	4.45%
20		GFY	1e-27	0.81%	0.44%
21		EHF	1e-26	6.92%	5.76%
22		GABPA	1e-25	6.68%	5.54%
23		ERG	1e-25	9.53%	8.19%
24		NRF	1e-24	2.78%	2.09%
25		ETV1	1e-23	9.22%	7.94%

Supplemental Figure 1. HOMER analysis showing overrepresented binding motifs for known transcription factors under open chromatin peaks at 3 developmental stages A) iPSC, B) LP3a, C) ALIw5.

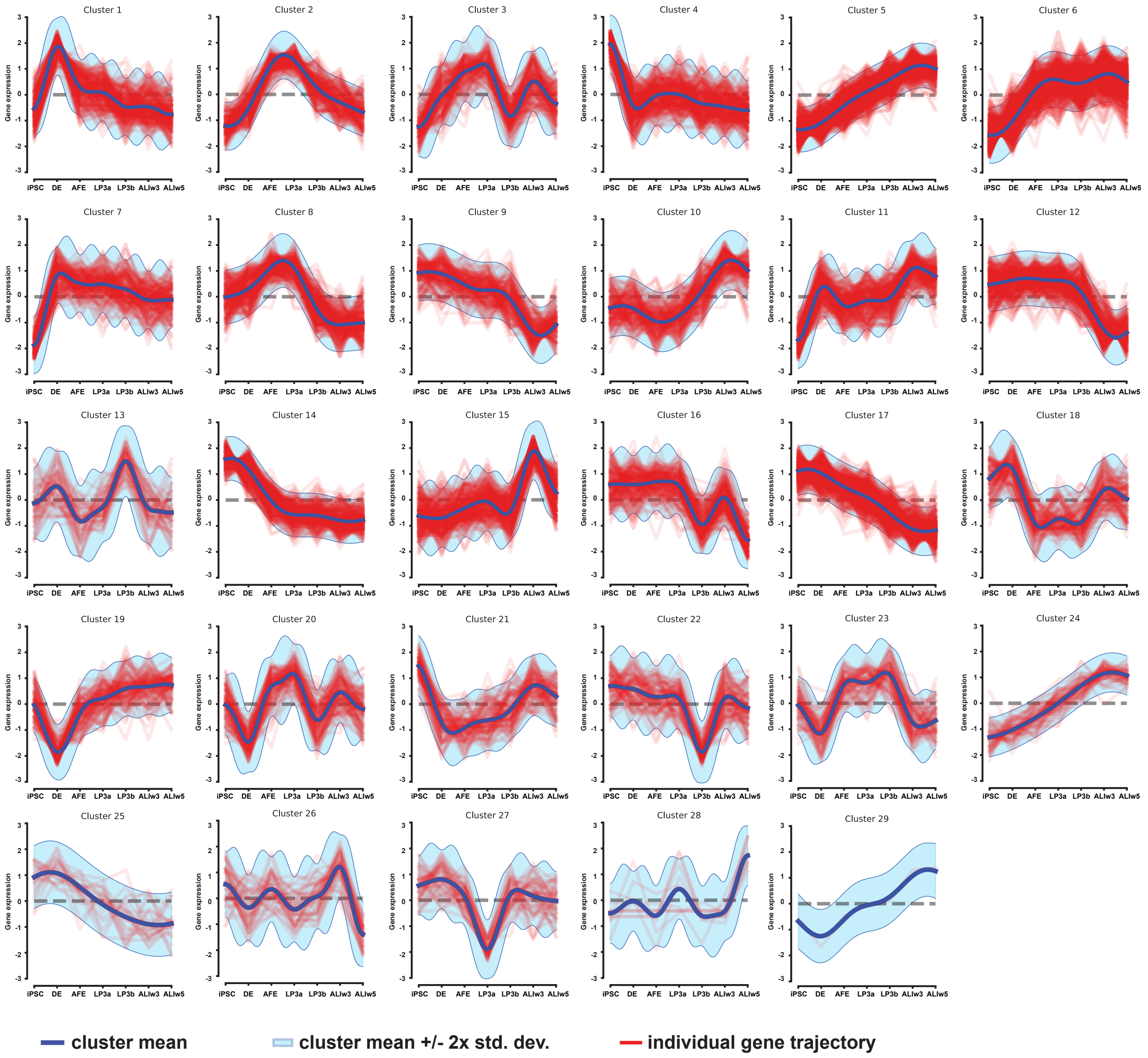

Supplemental Figure 2. Gene expression trajectories from iPSC to ALIw5 for iPSC line ND2.0. The expression profile for individual genes across the developmental pathway is stratified into cluster models with the aggregate mean and standard deviation plotted for each.

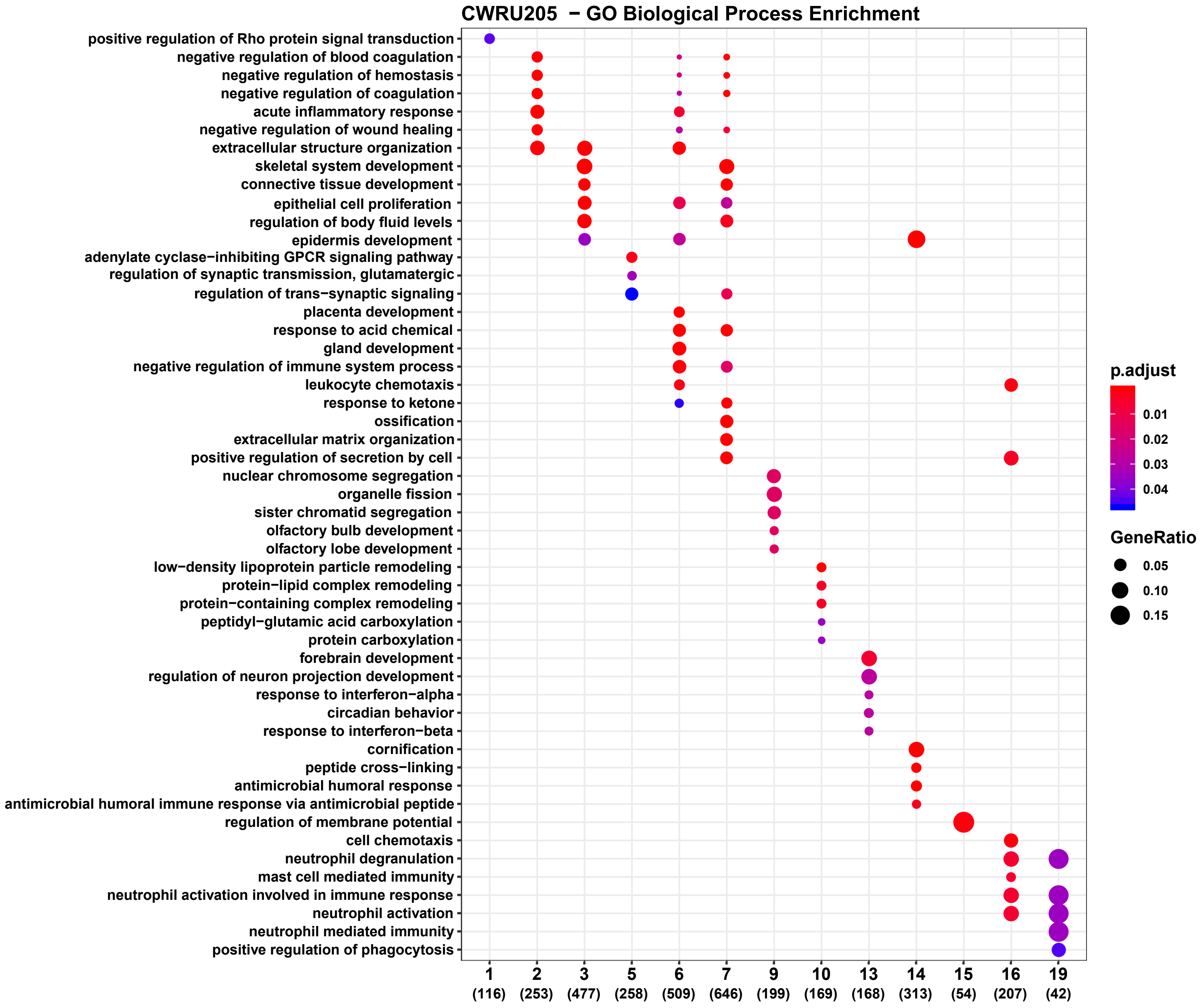

Supplemental Figure 3. Dot plot of GO biological process terms enriched in gene expression clusters identified in the differentiation of iPSC line CWRU205. Clustering was performed using an infinite Gaussian process mixture model. Only trajectories with statistically significant enrichment for a biological process category (13/21) are shown.

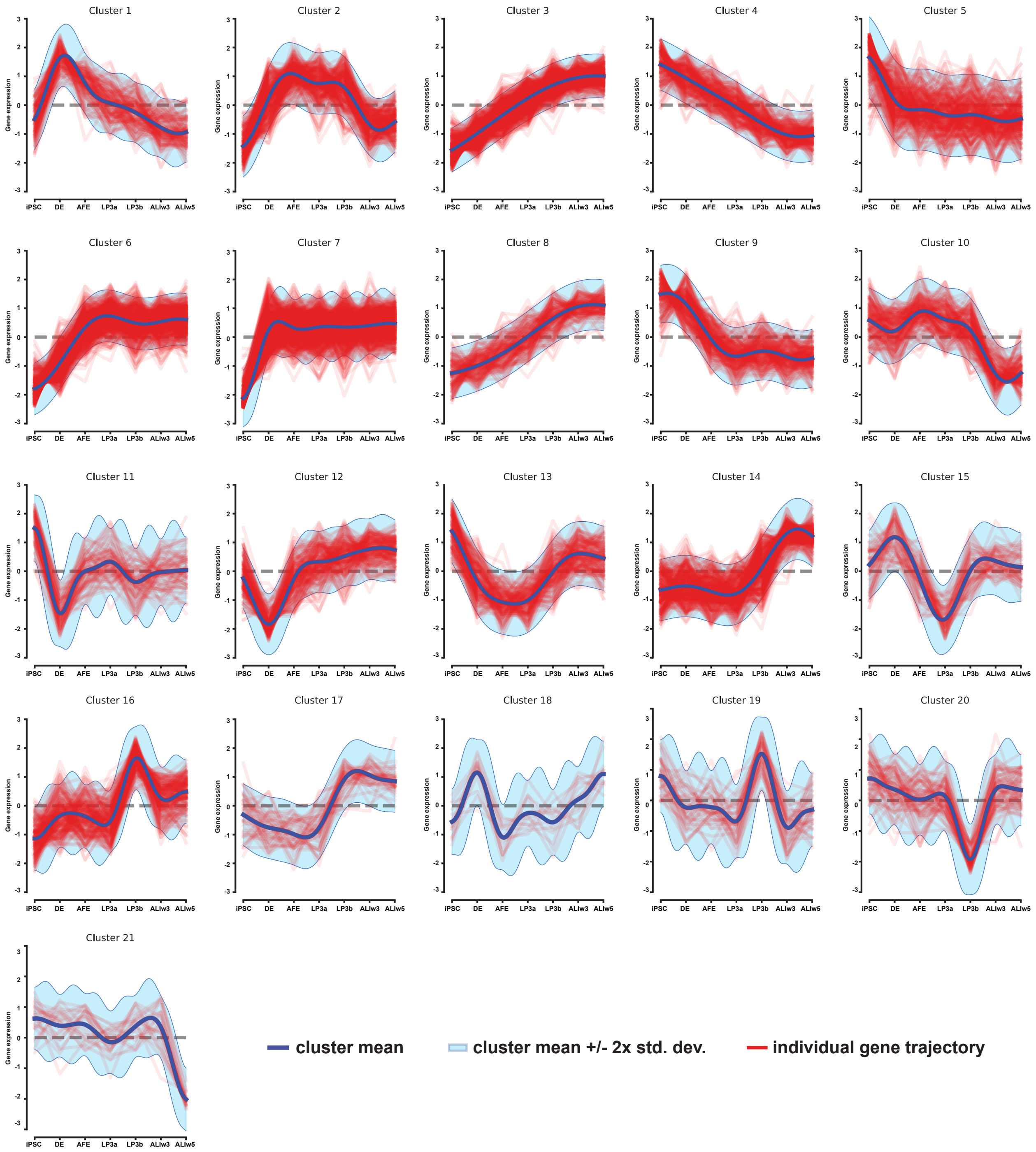

Supplemental Figure 4. Gene expression trajectories from iPSC to ALIw5 for iPSC line CWRU205. The expression profile for individual genes across the developmental pathway is stratified into cluster models with the aggregate mean and standard deviation plotted for each.

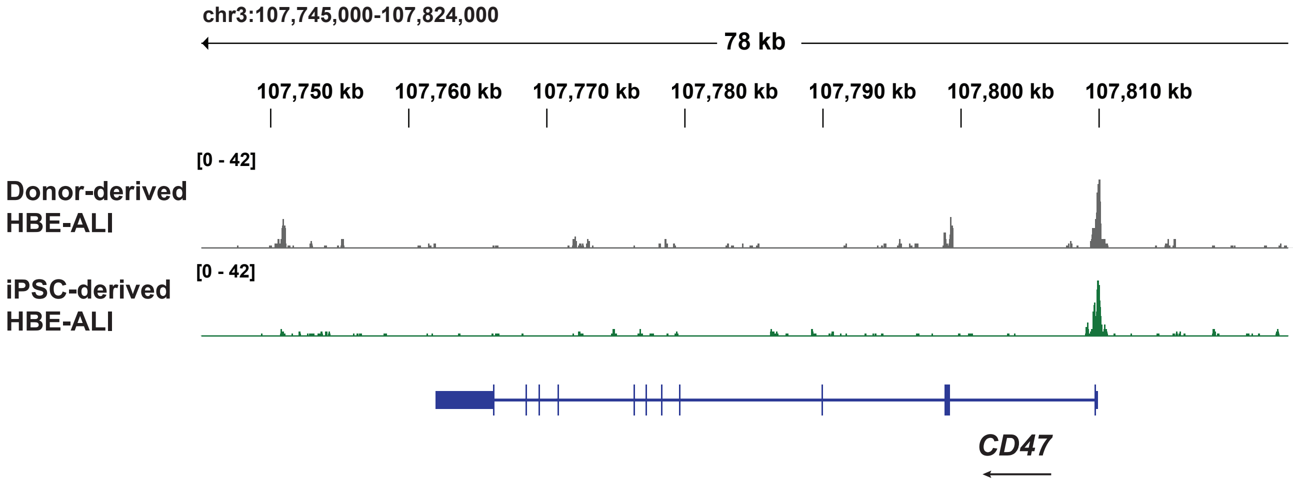

Supplemental Figure 5. UCSC genome browser graphic of ATAC-seq tracks (IDR) for donor-derived HBE-ALI and iPSC-derived ALI cultures at the *CD47* locus.

Supplementary Table 1. RT-qPCR primers used to validate iPSC to ALI differentiation.

SYBR primers	Sequence (5'>3'); Forward, Reverse	Reference	Stage Assayed
SOX2	GCACATGAAGGAGCACCCGGATTA<br>CGGGCAGCGTGTACTTATCCTTCTT	1	DE, AFE, LP3a, LP3b
SOX9	GAGGAAGTCGGTGAAGAACG<br>ATCGAAGGTCTCGATGTTGG	1	DE, AFE, LP3a, LP3b
SOX17*	AAGGGCGAGTCCCGTATC<br>TTGTAGTTGGGGTGGTCCTG	1	DE, AFE, LP3a, LP3b
FOXA2	AGGAGGAAAACGGGAAAGAA<br>CAACAACAGCAATGGAGGAG	1	DE, AFE
MUC5AC	CCATTGCTATTATGCCCTGTGT<br>TGGTGGACGGACAGTCACT	1	DE, AFE, LP3a, LP3b, ALIw3, ALIw5
MUC16	CCAGTCCTACATCTTCGGTTGT<br>AGGGTAGTTCCTAGAGGGAGTT	1	DE, AFE, LP3a, LP3b, ALIw3, ALIw5
FOXJ1*	GAGCGGCGCTTTCAAGAAG<br>GGCCTCGGTATTCACCGTC	1	DE, AFE, LP3a, LP3b, ALIw3, ALIw5
TP63	ACTGCCAAATTGCAAAGACA<br>TGACTAGGAGGGGCAATCTG	This study	DE, AFE, LP3a, LP3b, ALIw3, ALIw5
KRT5	GGAGTTGGACCAGTCAACATC<br>TGGAGTAGTAGCTTCCACTGC	1	DE, AFE, LP3a, LP3b, ALIw3, ALIw5
NKX2.1	ACCAGGACACCATGAGGAAC<br>CGCCGACAGGTACTTCTGTT	1	DE, AFE
PGK1	TAACAAGCTGACGCTGGAC<br>GCAGCCTTAATCCTCTGGTT	2	DE, AFE, LP3a, LP3b, ALIw3, ALIw5

1. Wong, A. P. et al. Efficient generation of functional CFTR-expressing airway epithelial cells from human pluripotent stem cells. Nat Protoc 10, 363-381, doi:10.1038/nprot.2015.021 (2015).
2. Mutolo, M.J. et al. A transcription factor network represses *CFTR* gene expression in airway epithelial cells. Biochem J 7, 1323-1334, doi: 10.1042/BCJ20180044 (2018).
*Figure 3B

Supplementary Table 2. Gene lists corresponding to expression clusters in Figure 4

A Cluster 2

ACAA1	COPZ2	GPBAR1	MT2A	SHBG
ACADL	COTL1	GPC5	MYBPC3	SLC1A3
ADAMTS6	CREB3L3	GPER1	MYH3	SLC2A9
ADAMTS16	CSDC2	GRM3	NAT8	SLC5A7
ADAMTSL4	CSPG5	GRM7	NCALD	SLC7A2
AFP	CSRP3	GSTA2	NKAIN4	SLC10A1
AGBL3	CXCL13	HAMP	NOL4	SLC16A10
AGXT2	DACT1	HMGCS2	NPTXR	SLC22A6
AHSG	DCDC1	HOXC8	OGDHL	SLC22A10
AKAP5	DCX	HOXD10	OR4E1	SLC23A1
AKAP14	DEPTOR	HPN	ORM1	SLC26A7
AKR1D1	DGAT2	HPX	PITX1	SLIT2
ALDH8A1	DIO1	IAPP	PKD2L1	SLN
ANGPTL3	DOK6	IQCJ-SCHIP1	PLBD1	SMAD9
ANKRD34C	DPP7	ITIH1	PLEKHF1	SNX31
ANKS1A	EBF4	ITIH3	PLG	SPTLC3
APOA4	EDIL3	KCNA5	PLN	SPTSSB
APOA5	ERVMER34-1	KCTD8	PLXNC1	SRGAP3
APOB	ERVV-2	KLB	PON1	STEAP1
ASGR2	FAM151A	KNG1	PPP1R3G	SULT2A1
ATF7IP2	FAM163A	LEAP2	PROC	TCF24
ATP6V0A4	FCGR2B	LECT2	PRSS35	TDRD6
BCL2	FGA	LGI4	PSG8	TENM1
BIN1	FGG	LGSN	RASD1	TF
C1QTNF7	FILIP1L	LPA	RBMS3	TIMD4
CA3	FMO1	LRRC25	RBP4	TIMP3
CAMK2B	FOXA1	LRRC63	RFX6	TMEM64
CCDC110	FRMD1	LRRTM3	RFX8	TMEM178A
CCDC154	FTCD	MAATS1	RS1	TNNT2
CCR5	G0S2	MANSC4	S100P	TPD52L1
CD3G	GABRB1	MAT1A	SCARA5	TUBA3E
CDH7	GALNT15	MBL2	SCML4	TUBB4A
CDH16	GCGR	MEOX1	SCN2A	TYRP1
CDKN1C	GIF	MID1	SEC14L5	UPK3A
CEBPA	GLDC	MOGAT3	SELPLG	VAT1L
CFC1	GLP1R	MROH8	SEMA3D	VIM
CHRNA2	GLTPD2	MS4A10	SERPINA12	WNT8B
CKMT2	GLYAT	MST1	SERPINC1	WNT11
CLEC10A	GLYATL1	MT1B	SERPIND1	XYLB
COL2A1	GNRH2	MT1G	SERPINF2	ZBED3
COL7A1	GOLGA6L2	MT1X	SGIP1	ZNF804B

B Cluster 5

AADAC	ATP8B1	CDKL1	DLK2	FOSL1	HR	KRT80	MR1
ABCA5	AZGP1	CDKN2A	DNAJC12	FRK	HRK	KRT81	MUC20
ABCA10	B3GALT1	CDRT1	DNAJC22	FRMD4B	HS3ST1	KRT86	MYBPC1
ABCB11	B3GALT2	CEACAM7	DNER	FRMD5	HSH2D	LACC1	MYH14
ABCC3	BAAT	CFHR3	DPYD	GALNT3	IFI6	LAMB3	N4BP2L1
ABHD2	BCAN	CFI	DRAM1	GBP1	IFIT5	LAMP2	NAALADL2
ABHD3	BCL3	CITED4	DST	GBP2	IFNAR1	LCOR	NALCN
ABLIM2	BCL10	CLDN9	DUOX2	GBP3	IGFBP7	LGALS3BP	NBEAL1
ABLIM3	BCO2	CLDN23	DUSP10	GC	IL2RG	LGALS8	NCCRP1
ACMSD	BICC1	CLEC2B	DYNLRB2	GCKR	IL6ST	LHFPL3	NCEH1
ACSL4	BIRC3	CLMN	EBI3	GCNT3	IL12A	LIF	NCMAP
ACSS1	BMP1	CMPK1	EDN3	GCSAM	IL15RA	LIPH	NEDD4
ACTBL2	BRINP2	CORO2B	EGLN1	GDA	IL17RE	LIPK	NEDD4L
ACTR3C	BTBD11	CP	EHF	GDPD5	IL18	LMO4	NEGR1
ADAM9	BTC	CPB2	ELF1	GFI1	IL31RA	LNPEP	NFATC2
ADAM12	BTG1	CPEB4	ELOVL7	GGT5	IL32	LONRF2	NFIB
ADAP1	BTNL3	CPM	ENAM	GJB4	IL36RN	LRG1	NFKB1
ADCY9	C3	CPT1A	ENDOD1	GK	INHBA	LRGUK	NIN
AHR	C4BPA	CREB5	EPB42	GLB1L2	IQCE	LRP10	NMI
ALPK1	CACNA1D	CTAGE1	EPDR1	GLP2R	IRAK2	LRRTM2	NMNAT3
ALS2CL	CACNA1H	CTSH	EPN3	GLS	ITGA3	LTBP3	NOX5
AMBP	CAMK2N1	CX3CL1	EPS8L1	GNG12	ITGAV	LTBR	NPAS2
AMY2B	CAPN3	CXCL1	EQTN	GOLGA7B	ITGB1	LURAP1L	NPC1L1
ANKRD22	CAPN6	CXCL2	EREG	GP2	ITM2B	LYPD6	NPFFR2
ANXA4	CARD10	CXCL3	ERP27	GPR37L1	ITPR2	MAFF	NR1H4
ANXA9	CARD11	CXCL5	ETNK1	GPR155	ITPR3	MAFK	NRAP
ANXA10	CASC1	CXCL11	ETV6	GPR157	JAG1	MAMLD1	NRIP1
ANXA13	CASP1	CYP1A1	EVI2A	GRHL2	JAK1	MAOA	NT5C1B
AP1S3	CASP4	CYP2B6	F5	GSAP	JUNB	MAP2	NXNL2
APCDD1	CAV2	CYP2C19	F12	GSDMA	KCNC3	MAP3K8	OLFM4
APCS	CBX6	CYP2J2	F13B	HDAC9	KCNJ15	MAPK11	ONECUT1
APOL2	CCL15	CYP4V2	FAM47E	HEBP1	KISS1	MBNL2	ONECUT3
ARAP2	CCL20	CYP26B1	FAM83E	HFE	KISS1R	MDGA1	PAH
ARHGAP8	CCL28	CYTH4	FAM102A	HID1	KLF3	MET	PAQR8
ARHGEF10L	CCPG1	DAPP1	FAM110C	HIST1H3E	KLF13	MFSD1	PARP8
ARHGEF12	CD28	DAZAP2	FAT1	HIST4H4	KLHL5	MFSD6	PARP9
ARHGEF38	CD58	DCHS2	FCRLA	HIVEP3	KLHL29	MICAL3	PCDHGA7
ARL14	CD59	DENND4A	FFAR2	HLA-DQB1	KLRC3	MISP	PCDHGB1
ARNTL2	CD69	DGKA	FGF14	HNF1A	KPNA7	MMP7	PDE3A
ART4	CDC42EP1	DGKH	FGF22	HNMT	KRT15	MPV17L	PDGFC
ATG2B	CDH1	DHRS7C	FHL2	HOGA1	KRT17	MPZL3	PDGFD

PDZD7	RAET1G	SLC9C1	STARD10	TOM1L2
PECR	RAI2	SLC10A5	STAT1	TOX
PFKFB3	RAP1GAP	SLC14A1	STAT3	TOX3
PGPEP1	RASSF5	SLC16A7	STC2	TP53I3
PHACTR3	RASSF9	SLC17A1	STX7	TREM1
PHLDA2	RBP2	SLC17A4	STXBP4	TRIM22
PI3	RDH12	SLC17A8	SUCNR1	TSHZ1
PIEZO1	RELL1	SLC26A9	SUV39H1	TTLL7
PIEZO2	RGPD1	SLC28A3	SV2B	TTLL11
PIGR	RHOBTB1	SLC30A1	SVIL	TWF1
PIK3C2G	RHOJ	SLC35G2	SYT5	UGCG
PIWIL4	RHOV	SLC38A1	SYT8	UGT2B4
PKHD1	RIC8B	SLC38A3	TACSTD2	UGT2B17
PLA1A	RNF13	SLC43A1	TANC2	UPK1B
PLAU	RNF103	SLC45A1	TANK	USH1C
PLEKHA4	RNF207	SMARCA2	TBC1D4	VGLL1
PLEKHG7	RNF223	SMCO2	TBC1D8	VGLL3
PLS1	RTN4RL2	SMIM5	TC2N	VIP
PLSCR4	RUNX1	SMIM22	TCN1	VMP1
PLXNB2	S100A6	SMOC1	TFAP2A	VNN1
PNKD	SAA2	SMPDL3B	TFPI2	VNN3
PON2	SAMD9	SOBP	TGFBR2	VTCN1
PPARG	SAMD13	SOD2	TIMP2	WDR78
PPIP5K1	SCTR	SORT1	TMBIM1	WNK2
PPP1R13B	SDC1	SOWAHA	TMEM62	WNT4
PRDM16	SDCBP2	SOX6	TMEM105	WNT7B
PRKAA1	SERPINA1	SP100	TMEM106B	WWC1
PRR5-ARHGAP8	SERPINA3	SP110	TMEM139	WWTR1
PRR22	SERPINA4	SPAG6	TMEM156	YY2
PRR26	SERPINB7	SPATA6L	TMEM169	ZBTB20
PRRG2	SERPINB8	SPATA8	TMEM184A	ZC3H12A
PRRG4	SERPINE1	SPATA12	TMEM217	ZC3H12C
PRSS22	SFRP5	SPATA13	TMOD2	ZPLD1
PTGER3	SFTPA2	SPINK1	TNFRSF10D	
PTGES	SGPP2	SPIRE1	TNFRSF12A	
PTHLH	SH2B3	SPNS2	TNFRSF14	
PTK2B	SH2D3A	SPP1	TNFSF13	
PTPRK	SH3TC2	SPTB	TNFSF15	
PTPRO	SHROOM3	SREBF1	TNIP1	
PYROXD1	SKAP2	SRPX2	TNNI2	
RAB27B	SLC2A13	ST7	TNS1	

C Cluster 17

ABCC12	BMP7	CHD1L	DOCK8	FOXI2	HIST1H1E	KLF15	NDC80	PDE4B	RCOR2	SMC4	TNNC2	ZNF611
ABHD12B	BRIP1	CHODL	DPEP3	FOXI3	HIST1H2AG	KLK7	NECAB2	PDE6B	RCSD1	SNAI1	TNR	ZNF614
ABRA	C1QTNF4	CHRNA3	DPPA2	FOXM1	HJURP	LAMA2	NEIL3	PDLIM3	RDH16	SNAP91	TOP2A	ZNF750
ACPP	CABP1	CHST6	DSCAM	FRZB	HMGA1	LCNL1	NEK2	PDZRN4	RECK	SNRNP25	TPX2	ZNF761
ADAM11	CACNA1I	CIT	DSCC1	FSCN1	HMGB2	LDB2	NGFR	PGM1	RFC4	SOCS2	TRABD2A	ZNF878
ADAM33	CACNA2D2	CKAP2L	DTL	FSD1	HSD17B14	LDLRAD3	NLGN1	PHOSPHO1	RHOH	SOHLH2	TRDN	ZSCAN1
ADAMTS14	CADM2	CLDN11	DTNB	FXYD6	HSPA12B	LIFR	NLRP3	PI15	RIPPLY3	SOX7	TRIM46	
ADCY1	CADM3	CLDN19	DUSP2	FZD4	HSPB7	LIG1	NLRP12	PIANP	RIT2	SOX12	TRPC4	
ADRA2B	CALML6	CLEC14A	E2F1	FZD7	HTR1E	LIMS2	NMRK2	PIK3R5	RMI2	SP8	TSPAN33	
AIF1L	CAND2	CLVS2	EDNRA	GABRG1	IFNE	LINGO2	NMU	PINLYP	RNASE1	SPAG5	TTK	
AKR1E2	CAP2	CNTD2	EFHD1	GABRR1	IGFBP6	LINGO3	NOG	PLD5	RRH	SPTA1	TTN	
ALG1L	CAPN11	CNTNAP1	EGF	GALNT13	IGFL4	LMNB1	NOTUM	PLK4	RTP1	SSTR2	TYMS	
AMPH	CASQ2	COCH	EID3	GAS1	IL6	LPAR4	NPIPA5	PLTP	RUNX1T1	ST6GAL1	TYROBP	
ANK1	CBLN1	COL4A6	EIF4E1B	GAS6	IL17RD	LRRN4	NPM2	PLXDC1	RYR1	ST6GALNAC2	UBE2S	
AP1S2	CCDC63	COL8A1	ELOVL4	GINS2	INMT	LRRTM4	NPR1	PODXL	S1PR3	ST8SIA4	UGT3A2	
APOE	CCDC81	COL9A1	EMILIN3	GIPC3	IQGAP3	LRTM1	NR6A1	POLD1	S100Z	ST8SIA6	UPK1A	
ARHGAP11A	CCKBR	COL23A1	ENHO	GJA1	ISM2	LURAP1	NRG2	POLE2	SALL4	STARD9	USP51	
ARHGAP22	CCNA2	COLEC11	ENPP2	GLB1L3	ITGA2B	MAD2L1	NRP2	POMC	SCGB3A2	SUPT3H	VAX1	
ARHGAP28	CCNB1	CPVL	EPB41L3	GLDN	ITK	MAD2L2	NRROS	POPDC3	SEMA5B	SYCE2	VBP1	
ARHGAP33	CCNE1	CPZ	EPHX3	GLI2	ITLN2	MAGEB17	NT5DC2	PPP1R16B	SEPT5	SYN2	VRTN	
ARHGDIG	CCNG1	CRHBP	EPPIN	GLIS1	ITM2A	MARC1	NT5M	PRC1	SERPINE2	SYNDIG1	WDR62	
ARHGEF6	CCR10	CRIP3	ERBB4	GMPR	ITPRIP	MARCH11	NUMBL	PRIMA1	SGSM1	SYT2	WDR76	
ARHGEF15	CD34	CRISPLD1	ERCC6L	GNAO1	JARID2	MATN3	NUSAP1	PROB1	SH3BP5	SYT17	WDR86	
ARID3B	CD79B	CRX	ERMN	GNG2	KCNAB2	MCF2	OIP5	PROCA1	SH3GL2	TBX6	WFDC1	
ARL11	CD163	CRYGN	ESCO2	GNG11	KCNE3	MCM10	OLFML2A	PROKR1	SH3RF3	TCEAL2	WFDC2	
ARMCX2	CDC6	CST2	ESPL1	GNG13	KCNH1	MDK	OR51B5	PRPH2	SHANK1	TCEAL5	XKR8	
ART5	CDC7	CST4	EXO1	GNGT2	KCNRG	MEIS3	OR51E2	PRRG3	SHCBP1	TDRD5	XRCC2	
ASF1B	CDC42EP3	CTHRC1	FABP7	GPR63	KCTD14	MKI67	OR51M1	PTGDR	SHE	TEKT3	ZAP70	
ASIC2	CDC45	CTSV	FAM13C	GPR83	KCTD19	MLC1	OTX2	PTP4A3	SKA3	TET1	ZBBX	
ASRGL1	CDH13	DBF4B	FAM43B	GPRASP1	KIF2C	MND1	OVCH2	PTPN13	SKIDA1	TEX19	ZCCHC12	
ASTN1	CDH15	DCLK3	FAM49A	GREM2	KIF4A	MRAP2	OXER1	PYCR1	SKP2	THEMIS2	ZFP42	
ATAD5	CDK1	DCT	FAM89A	GRID2	KIF4B	MRC2	OXTR	PYGM	SLC1A6	THSD7B	ZFR2	
ATP1A2	CDKN3	DEPDC1	FAM111B	GRIK3	KIF7	MSH6	PALD1	PYY	SLC2A4	TICRR	ZIC3	
ATP1A4	CDT1	DHRS13	FAM124A	GRK1	KIF11	MSI1	PAMR1	RAB39A	SLC7A3	TIPIN	ZIC5	
AURKA	CELF2	DIAPH3	FAM126A	GRM1	KIF14	MYBL2	PAQR4	RAD51	SLC8A1	TLN2	ZNF90	
AURKB	CELF5	DLG2	FAM189A1	GTSE1	KIF17	MYF6	PARVB	RAD54B	SLC12A8	TMEM179	ZNF93	
B3GALNT1	CENPF	DLGAP3	FGF13	GUCY2D	KIF18A	MYLK2	PARVG	RAD54L	SLC16A14	TMEM200B	ZNF280A	
BCAT2	CENPH	DNA2	FN3K	GYPE	KIF20A	MYRIP	PAX2	RADIL	SLC22A17	TMEM221	ZNF331	
BCL6B	CENPI	DNAJB5	FNDC5	HAUS4	KIF25	NANOG	PBK	RASIP1	SLC24A2	TMEM240	ZNF521	
BEND4	CENPK	DNAJC6	FOXG1	HBE1	KIF26A	NAP1L2	PCDH8	RBM20	SLC35F3	TMOD1	ZNF569	
BLM	CEP19	DOC2A	FOXH1	HELLS	KLF8	NCAN	PCDH10	RBM46	SLITRK5	TNNC1	ZNF578	

Suppl.Table 3 Gene ontology biological process enrichment of DE-specific differentially expressed genes.

Term_name	Term_id	Adj *p* Value	-Log10(*p*)	Term size	Query	Intersection
anatomical structure morphogenesis	GO:0009653	4.16E-08	7.380905998	2677	721	176
cellular developmental process	GO:0048869	3.26E-07	6.487043529	4356	721	252
system development	GO:0048731	3.76E-07	6.42430811	4849	721	274
axon guidance	GO:0007411	8.66E-07	6.062269062	270	721	36
neuron projection guidance	GO:0097485	9.61E-07	6.017130471	271	721	36
cell differentiation	GO:0030154	1.01E-06	5.996379586	4162	721	241
cell development	GO:0048468	3.63E-06	5.440322581	2139	721	142
animal organ development	GO:0048513	4.67E-06	5.331127487	3533	721	209
neuron differentiation	GO:0030182	8.51E-06	5.070083076	1356	721	100
axon development	GO:0061564	3.75E-05	4.426175852	504	721	49
regulation of developmental process	GO:0050793	5.66E-05	4.246999325	2641	721	162
axonogenesis	GO:0007409	5.78E-05	4.23815098	464	721	46
generation of neurons	GO:0048699	6.28E-05	4.202170515	1505	721	105
chemotaxis	GO:0006935	9.13E-05	4.039403198	632	721	56
taxis	GO:0042330	0.000101748	3.992475098	634	721	56
nervous system development	GO:0007399	0.000121035	3.91709031	2340	721	146
neurogenesis	GO:0022008	0.000122461	3.912001897	1602	721	109
regulation of multicellular organismal development	GO:2000026	0.00018452	3.733956407	2091	721	133
regulation of multicellular organismal process	GO:0051239	0.000309565	3.509247981	3132	721	182
cell morphogenesis involved in neuron differentiation	GO:0048667	0.000533153	3.273148036	582	721	51
regionalization	GO:0003002	0.00141363	2.849664252	343	721	35
pattern specification process	GO:0007389	0.001650698	2.782332463	439	721	41
cell morphogenesis involved in differentiation	GO:0000904	0.002478892	2.605742439	734	721	58
neuron projection morphogenesis	GO:0048812	0.002887731	2.539443258	650	721	53
cellular component morphogenesis	GO:0032989	0.004166279	2.38025169	1133	721	79

Suppl. Table 4. Gene ontology biological process enrichment of AFE-specific differentially expressed genes.

term_name	term_id	Adj *p* Value	-Log10(*p*)	Term Size	Query	Intersection
extracellular structure organization	GO:0043062	2.15E-40	39.66810429	405	1191	117
extracellular matrix organization	GO:0030198	4.31E-39	38.36572998	351	1191	107
cell adhesion	GO:0007155	6.91E-31	30.16068423	1399	1191	219
biological adhesion	GO:0022610	1.65E-30	29.78315828	1407	1191	219
anatomical structure morphogenesis	GO:0009653	1.98E-30	29.70250176	2677	1191	337
tube morphogenesis	GO:0035239	1.48E-28	27.82888362	922	1191	164
tube development	GO:0035295	2.61E-28	27.58339441	1112	1191	184
system development	GO:0048731	1.09E-27	26.96283997	4849	1191	505
circulatory system development	GO:0072359	2.31E-25	24.63658624	1162	1191	183
tissue development	GO:0009888	2.32E-25	24.63423324	1995	1191	263
blood vessel development	GO:0001568	4.96E-24	23.30413989	766	1191	138
cardiovascular system development	GO:0072358	4.59E-23	22.33850965	810	1191	141
vasculature development	GO:0001944	4.65E-23	22.33269022	801	1191	140
animal organ morphogenesis	GO:0009887	1.01E-22	21.99680308	1004	1191	161
cell differentiation	GO:0030154	3.75E-22	21.42562618	4162	1191	434
anatomical structure formation (morphogenesis)	GO:0048646	7.38E-22	21.13201876	1180	1191	177
cell surface receptor signaling pathway	GO:0007166	3.56E-21	20.44867934	3051	1191	342
tissue morphogenesis	GO:0048729	1.01E-20	19.99774662	627	1191	116
blood vessel morphogenesis	GO:0048514	1.72E-20	19.76503547	684	1191	122
cellular developmental process	GO:0048869	2.03E-20	19.69347695	4356	1191	443
regulation of signaling	GO:0023051	1.02E-19	18.9916595	3568	1191	379
regulation of cell communication	GO:0010646	3.53E-19	18.45244874	3541	1191	375
skeletal system development	GO:0001501	6.55E-19	18.18400284	518	1191	100
regulation of multicellular organismal process	GO:0051239	7.65E-19	18.11645089	3132	1191	341
animal organ development	GO:0048513	8.73E-19	18.05895955	3533	1191	373
locomotion	GO:0040011	9.32E-19	18.03044494	1940	1191	240
regulation of cell adhesion	GO:0030155	2.99E-18	17.5242347	679	1191	117
cell migration	GO:0016477	3.05E-18	17.51619661	1557	1191	204
response to organic substance	GO:0010033	1.25E-17	16.90200892	3233	1191	345
epithelium development	GO:0060429	2.43E-17	16.61495805	1241	1191	172
negative regulation of multicellular process	GO:0051241	3.70E-17	16.43182844	1288	1191	176
cell motility	GO:0048870	9.20E-17	16.03643145	1713	1191	214
localization of cell	GO:0051674	9.20E-17	16.03643145	1713	1191	214
regulation of multicellular development	GO:2000026	1.08E-16	15.96491018	2091	1191	247
regulation of developmental process	GO:0050793	1.52E-16	15.81818934	2641	1191	293
cellular response to organic substance	GO:0071310	1.79E-16	15.74655138	2656	1191	294
embryo development	GO:0009790	4.17E-16	15.38025399	1005	1191	146
response to endogenous stimulus	GO:0009719	1.58E-15	14.80239804	1664	1191	206
regulation of localization	GO:0032879	2.06E-15	14.68633349	2749	1191	298
regulation of biological quality	GO:0065008	2.31E-15	14.63673699	3911	1191	390

morphogenesis of an epithelium	GO:0002009	3.15E-15	14.50162061	491	1191	90
movement of cell or subcellular component	GO:0006928	3.39E-15	14.46954109	2170	1191	249
cellular response to chemical stimulus	GO:0070887	5.54E-15	14.25647709	3202	1191	333
regulation of cell differentiation	GO:0045595	6.53E-15	14.18490762	1821	1191	218
nervous system development	GO:0007399	8.62E-15	14.06452209	2340	1191	262
angiogenesis	GO:0001525	1.05E-14	13.97858509	590	1191	100
enzyme linked receptor protein signaling pathway	GO:0007167	1.07E-14	13.97251072	1041	1191	146
embryonic morphogenesis	GO:0048598	1.34E-14	13.87403272	592	1191	100
negative regulation of developmental process	GO:0051093	1.55E-14	13.80870917	1035	1191	145
cell population proliferation	GO:0008283	1.75E-14	13.75687165	2127	1191	243
cell development	GO:0048468	3.54E-14	13.45082724	2139	1191	243
regulation of cell migration	GO:0030334	4.51E-14	13.34589954	924	1191	133
regulation of signal transduction	GO:0009966	1.09E-13	12.96234916	3226	1191	330
mesenchyme development	GO:0060485	1.11E-13	12.95392635	274	1191	61
regulation of anatomical structure morphogenesis	GO:0022603	1.34E-13	12.87355865	1113	1191	150
embryonic organ development	GO:0048568	1.52E-13	12.81835702	432	1191	80
urogenital system development	GO:0001655	1.74E-13	12.76067458	332	1191	68
cardiac chamber development	GO:0003205	1.92E-13	12.71618657	168	1191	46
cellular response to endogenous stimulus	GO:0071495	2.56E-13	12.59099411	1403	1191	176
regulation of locomotion	GO:0040012	2.84E-13	12.54688468	1059	1191	144
regulation of cellular component movement	GO:0051270	2.95E-13	12.52983497	1070	1191	145
heart development	GO:0007507	3.32E-13	12.47900306	573	1191	95
cell-cell signaling	GO:0007267	4.17E-13	12.37947048	1544	1191	188
epithelial tube morphogenesis	GO:0060562	4.82E-13	12.31715259	322	1191	66
cellular response to growth factor stimulus	GO:0071363	5.92E-13	12.22770357	702	1191	108
mesenchymal cell differentiation	GO:0048762	5.99E-13	12.22228333	215	1191	52
regulation of cell motility	GO:2000145	6.33E-13	12.19878587	985	1191	136
response to growth factor	GO:0070848	6.34E-13	12.197646	732	1191	111
regulation of cell population proliferation	GO:0042127	8.08E-13	12.09256943	1702	1191	201
ossification	GO:0001503	8.55E-13	12.06782772	384	1191	73
cardiac chamber morphogenesis	GO:0003206	1.12E-12	11.95078885	129	1191	39
renal system development	GO:0072001	3.17E-12	11.49865183	293	1191	61
cardiac septum development	GO:0003279	3.28E-12	11.48359045	108	1191	35
morphogenesis of a branching epithelium	GO:0061138	6.78E-12	11.16900569	183	1191	46
heart morphogenesis	GO:0003007	7.08E-12	11.14977451	258	1191	56
cell-substrate adhesion	GO:0031589	1.09E-11	10.96349358	342	1191	66
response to chemical	GO:0042221	1.41E-11	10.85045963	4678	1191	432
neurogenesis	GO:0022008	1.69E-11	10.77201964	1602	1191	188
regulation of response to stimulus	GO:0048583	1.79E-11	10.7462775	4282	1191	402
kidney development	GO:0001822	3.37E-11	10.47193442	275	1191	57
positive regulation of cell communication	GO:0010647	6.00E-11	10.22190615	1786	1191	202

positive regulation of cell population proliferation	GO:0008284	6.24E-11	10.20495961	968	1191	129
ventricular septum development	GO:0003281	6.61E-11	10.17987905	71	1191	27
ameboidal-type cell migration	GO:0001667	9.39E-11	10.02711188	455	1191	77
morphogenesis of a branching structure	GO:0001763	1.30E-10	9.887133263	197	1191	46
regulation of molecular function	GO:0065009	1.33E-10	9.87555052	3220	1191	317
negative regulation of cell differentiation	GO:0045596	1.37E-10	9.862352161	739	1191	106
positive regulation of signaling	GO:0023056	1.62E-10	9.789896792	1792	1191	201
stem cell differentiation	GO:0048863	2.54E-10	9.594342561	208	1191	47
cartilage development	GO:0051216	2.54E-10	9.594342561	208	1191	47
cardiac septum morphogenesis	GO:0060411	3.18E-10	9.497938516	75	1191	27
branching morphogenesis of an epithelial tube	GO:0048754	3.44E-10	9.462927806	151	1191	39
positive regulation of cellular movement	GO:0051272	3.94E-10	9.404079539	562	1191	87

Suppl. Table 5. Gene ontology biological process enrichment of ALIw3-specific differentially expressed genes.

Term_name	Term_id	Adj *p* Value	-Log10(*p*)	Term Size	Query	Intersection
defense response	GO:0006952	3.00E-15	14.52342724	1726	366	93
response to other organism	GO:0051707	8.65E-15	14.06316654	1507	366	85
response to external biotic stimulus	GO:0043207	9.38E-15	14.02762039	1509	366	85
response to biotic stimulus	GO:0009607	2.28E-14	13.64121292	1531	366	85
hormone metabolic process	GO:0042445	9.99E-11	10.00059868	231	366	28
cornification	GO:0070268	2.49E-10	9.604564467	111	366	20
regulation of hormone levels	GO:0010817	5.84E-10	9.233253498	528	366	41
steroid metabolic process	GO:0008202	6.64E-10	9.17815427	328	366	32
cellular hormone metabolic process	GO:0034754	1.36E-09	8.867198772	121	366	20
inflammatory response	GO:0006954	3.65E-09	8.438123594	740	366	48
terpenoid metabolic process	GO:0006721	2.79E-08	7.554399881	111	366	18
innate immune response	GO:0045087	2.83E-08	7.547862229	985	366	55
defense response to other organism	GO:0098542	4.29E-08	7.367987068	1178	366	61
organic hydroxy compound metabolic process	GO:1901615	5.42E-08	7.266178094	531	366	38
retinoic acid metabolic process	GO:0042573	1.12E-07	6.951430149	26	366	10
keratinocyte differentiation	GO:0030216	3.53E-07	6.452460105	301	366	27
skin development	GO:0043588	3.88E-07	6.411660176	417	366	32
isoprenoid metabolic process	GO:0006720	4.78E-07	6.320290606	131	366	18
regulation of defense response	GO:0031347	1.04E-06	5.982955058	783	366	45
response to bacterium	GO:0009617	1.43E-06	5.845500822	705	366	42
retinoid metabolic process	GO:0001523	1.60E-06	5.795596783	93	366	15
regulation of response to external stimulus	GO:0032101	1.67E-06	5.776738631	1098	366	55
primary alcohol metabolic process	GO:0034308	1.96E-06	5.707823406	80	366	14
epidermal cell differentiation	GO:0009913	3.47E-06	5.459215208	357	366	28
diterpenoid metabolic process	GO:0016101	3.95E-06	5.403723995	99	366	15
retinol metabolic process	GO:0042572	4.46E-06	5.350998832	36	366	10
monocarboxylic acid metabolic process	GO:0032787	4.83E-06	5.315971577	649	366	39
epidermis development	GO:0008544	6.54E-06	5.184286592	467	366	32
antibiotic metabolic process	GO:0016999	8.72E-06	5.059487857	121	366	16
antimicrobial humoral response	GO:0019730	1.25E-05	4.902199069	124	366	16
positive regulation of defense response	GO:0031349	1.67E-05	4.776839661	512	366	33
alcohol metabolic process	GO:0006066	1.70E-05	4.770361659	359	366	27
positive regulation of response to external stimulus	GO:0032103	3.59E-05	4.445429188	640	366	37
lipid metabolic process	GO:0006629	5.07E-05	4.294866639	1412	366	61
progesterone metabolic process	GO:0042448	5.66E-05	4.247437041	17	366	7
keratinization	GO:0031424	9.02E-05	4.044721331	223	366	20

Supplementary Table 6

A. Gene ontology process enrichment analysis of genes upregulated at ALI in donor-derived HBE compared to iPSC-derived cells.

GO: Biological Process	Term ID	Adj *p* Value	-Log10(*p*)	Term size	Query	Intersection
cilium assembly	GO:0060271	4.70E-34	33.32815	369	1867	129
cilium movement	GO:0003341	6.28E-29	28.20196	73	1867	50
plasma membrane bounded cell projection assembly	GO:0120031	1.23E-26	25.90989	562	1867	153
cell projection assembly	GO:0030031	1.68E-26	25.77464	575	1867	155
microtubule-based movement	GO:0007018	2.10E-25	24.67851	289	1867	100
axoneme assembly	GO:0035082	3.22E-23	22.4925	63	1867	42
microtubule-based process	GO:0007017	5.18E-22	21.28566	778	1867	179
microtubule bundle formation	GO:0001578	2.43E-18	17.61483	95	1867	47
cell projection organization	GO:0030030	2.56E-18	17.59248	1578	1867	284
plasma membrane bounded cell projection organization	GO:0120036	1.52E-16	15.81728	1539	1867	273
organelle assembly	GO:0070925	2.92E-14	13.53477	847	1867	170
axonemal dynein complex assembly	GO:0070286	2.21E-13	12.65621	31	1867	23
cilium-dependent cell motility	GO:0060285	8.26E-13	12.08316	118	1867	46
cilium or flagellum-dependent cell motility	GO:0001539	8.26E-13	12.08316	118	1867	46
epithelial cilium movement involved in extracellular fluid movement	GO:0003351	2.52E-11	10.59923	27	1867	20
microtubule cytoskeleton organization	GO:0000226	6.69E-11	10.17484	576	1867	121
extracellular transport	GO:0006858	6.24E-10	9.204538	30	1867	20
epidermis development	GO:0008544	3.53E-09	8.452279	473	1867	101
cytoskeleton organization	GO:0007010	1.47E-08	7.831529	1371	1867	222
microtubule-based transport	GO:0099111	4.09E-08	7.388663	191	1867	53
cornification	GO:0070268	4.68E-08	7.329766	112	1867	38
motile cilium assembly	GO:0044458	6.13E-08	7.212746	23	1867	16
epithelium development	GO:0060429	9.59E-08	7.018208	1319	1867	212
outer dynein arm assembly	GO:0036158	1.90E-07	6.720248	16	1867	13
intraciliary transport	GO:0042073	2.53E-07	6.596435	53	1867	24
skin development	GO:0043588	3.60E-06	5.443374	420	1867	85

B. Gene ontology process enrichment analysis of genes upregulated at ALI in iPSC-derived compared to donor-derived HBE cells.

GO: Biological Process	Term ID	Adj *p* Value	-Log10(*p*)	Term size	Query	Intersection
biological adhesion	GO:0022610	1.04E-24	23.98228	1447	1778	275
cell adhesion	GO:0007155	1.09E-24	23.9635	1440	1778	274
homophilic cell adhesion via plasma membrane adhesion molecules	GO:0007156	1.06E-21	20.97362	167	1778	67
cell-cell adhesion via plasma-membrane adhesion molecules	GO:0098742	6.62E-21	20.1791	275	1778	88
cell-cell adhesion	GO:0098609	4.63E-15	14.33406	866	1778	169
extracellular matrix organization	GO:0030198	6.40E-11	10.19364	375	1778	87
extracellular structure organization	GO:0043062	7.54E-11	10.1226	376	1778	87
steroid metabolic process	GO:0008202	2.42E-10	9.616949	332	1778	79
negative regulation of coagulation	GO:0050819	2.51E-10	9.60014	58	1778	28
enzyme linked receptor protein signaling pathway	GO:0007167	4.49E-10	9.347358	1076	1778	182
hormone metabolic process	GO:0042445	5.11E-10	9.291464	244	1778	64
regulation of coagulation	GO:0050818	7.41E-09	8.130157	87	1778	33
regulation of hormone levels	GO:0010817	2.94E-08	7.531423	542	1778	105
negative regulation of blood coagulation	GO:0030195	4.93E-08	7.307361	52	1778	24
animal organ morphogenesis	GO:0009887	5.37E-08	7.270119	1095	1778	177
cellular component morphogenesis	GO:0032989	6.30E-08	7.200506	1177	1778	187
taxis	GO:0042330	6.48E-08	7.188624	651	1778	119
negative regulation of hemostasis	GO:1900047	8.16E-08	7.088267	53	1778	24
chemotaxis	GO:0006935	1.12E-07	6.950278	649	1778	118
cell-cell signaling	GO:0007267	1.33E-07	6.875175	1699	1778	249
cell morphogenesis	GO:0000902	1.44E-07	6.842883	1068	1778	172
synapse assembly	GO:0007416	2.78E-07	6.555755	180	1778	48
terpenoid metabolic process	GO:0006721	3.33E-07	6.477723	119	1778	37
regulation of blood coagulation	GO:0030193	5.50E-07	6.259398	80	1778	29
primary alcohol metabolic process	GO:0034308	5.90E-07	6.229063	90	1778	31
regulation of hemostasis	GO:1900046	7.76E-07	6.109868	81	1778	29
